# Standing genetic variation fuels rapid evolution of herbicide resistance in blackgrass

**DOI:** 10.1073/pnas.2206808120

**Published:** 2023-04-12

**Authors:** Sonja Kersten, Jiyang Chang, Christian D. Huber, Yoav Voichek, Christa Lanz, Timo Hagmaier, Patricia Lang, Ulrich Lutz, Insa Hirschberg, Jens Lerchl, Aimone Porri, Yves Van de Peer, Karl Schmid, Detlef Weigel, Fernando A. Rabanal

**Affiliations:** ^a^Institute of Plant Breeding, Seed Science and Population Genetics, University of Hohenheim, 70599 Stuttgart, Germany; ^b^Department of Molecular Biology, Max Planck Institute for Biology Tübingen, 72076 Tübingen, Germany; ^c^Department of Plant Biotechnology and Bioinformatics, Ghent University, 9052 Ghent, Belgium; ^d^Center for Plant Systems Biology, VIB, 9052 Ghent, Belgium; ^e^Department of Biology, The Eberly College of Science, Penn State University, State College, PA 16801; ^f^Gregor Mendel Institute, Austrian Academy of Sciences, Vienna Bio Center, 1030 Vienna, Austria; ^g^Friedrich Miescher Laboratory 72076 Tübingen, Germany; ^h^Agricultural Research Station, BASF SE, 67117 Limburgerhof, Germany; ^i^Department of Biochemistry, Genetics and Microbiology, University of Pretoria, Pretoria 0028, South Africa; ^j^College of Horticulture, Academy for Advanced Interdisciplinary Studies, Nanjing Agricultural University, Nanjing 210095, China

**Keywords:** *Alopecurus myosuroides*, herbicide resistance, rapid adaptation, blackgrass

## Abstract

Repeated herbicide applications in agricultural fields exert strong selection on weeds such as blackgrass (*Alopecurus myosuroides*), which is a major threat for temperate climate cereal crops. This inadvertent selection pressure provides an opportunity for investigating the underlying genetic mechanisms and evolutionary processes of rapid adaptation, which can occur both through mutations in the direct targets of herbicides and through changes in other, often metabolic, pathways, known as non-target-site resistance. How much target-site resistance (TSR) relies on de novo mutations vs. standing variation is important for developing strategies to manage herbicide resistance. We first generated a chromosome-level reference genome for *A. myosuroides* for population genomic studies of herbicide resistance and genome-wide diversity across Europe in this species. Next, through empirical data in the form of highly accurate long-read amplicons of alleles encoding acetyl-CoA carboxylase (ACCase) and acetolactate synthase (ALS) variants, we showed that most populations with resistance due to TSR mutations—23 out of 27 and six out of nine populations for *ACCase* and *ALS*, respectively—contained at least two TSR haplotypes, indicating that soft sweeps are the norm. Finally, through forward-in-time simulations, we inferred that TSR is likely to mainly result from standing genetic variation, with only a minor role for de novo mutations.

## Significance

Because herbicides are designed to kill weeds, spontaneous mutants that are resistant to herbicide application have an enormous selective advantage and will often come to quickly dominate weed populations. While this is a nuisance for farmers, it provides opportunities for investigating in detail how organisms rapidly respond to strong selection, especially what role newly arising mutations play vs. mutations that are already present in a population. We first assembled a reference genome for blackgrass, the most economically damaging herbicide-resistant weed in Europe, and then combined analyses of known herbicide-resistant loci with forward-in-time simulations to show that target-site resistance mutations likely often predate the application of herbicides.

The agricultural use of herbicides has inadvertently selected for many herbicide-resistant grass weeds over the past several decades. Among these, blackgrass (*Alopecurus myosuroides*) has become the most economically damaging herbicide-resistant weed in Europe (1, 2). In England alone, the annual cost of resistance was estimated to be £0.4 billion (€ 0.47 billion) in lost gross profit (3).

We distinguish two resistance mechanisms. First, there is target-site resistance (TSR), which is caused by coding sequence mutations in or amplification of the genes encoding the proteins targeted by herbicides (4–9). Second, there is non-TSR (NTSR), which is associated with enhanced metabolic processes such as herbicide detoxification or sequestration (6, 10). To better understand how either type of resistance arises and may potentially come to dominate *A. myosuroides* populations, we need to learn more about the population structure and genetic diversity of the species across Europe. Previous regional studies have only found weak, if any, population structure, suggesting a very rapid and recent spread of the species (11, 12).

Two important drivers of the modes of evolution of herbicide resistance are the genetic architecture of the trait and the types of mutations that can give rise to it. TSR is conferred by mutations in single genes, with only a very small number of coding sequence changes allowing for herbicide resistance without eliminating the activity of the targeted protein. As in many other weeds, TSR in *A. myosuroides* has increased rapidly (13, 14), and as a consequence, herbicides that inhibit the action of acetolactate synthase (ALS, also known as acetohydroxyacid synthase) and acetyl-CoA carboxylase (ACCase) have widely lost their efficacy as weed control agents. In contrast, several different gene families, encoding detoxifying enzymes and transporters such as cytochrome P450 monooxygenases, glutathione S-transferases, ATP-binding cassette, and MFS-type transporters as well as glycosyltransferases, have been found to contribute to NTSR (reviewed in ref. 15). NTSR now accounts for a substantial proportion of resistance in agricultural fields and is becoming a major focus of herbicide resistance research (13).

Finally, the rapid speed with which herbicide resistance spreads in individual weed species raises the question of whether this is primarily due to repeated selection for rare de novo mutations, or more commonly arising from standing genetic variation, with herbicide-resistant alleles segregating in the population already before the widespread adoption of herbicide application. An optimal framework for distinguishing between these hypotheses is provided by forward-in-time genetic simulations (16).

To enable a better understanding of herbicide resistance evolution in *A. myosuroides*, we have generated a high-quality reference genome with PacBio long reads. Genotyping with double-digest restriction-site associated DNA (ddRAD) sequencing markers in 47 European field populations revealed considerable geographical population structure along with high effective population sizes. To characterize TSR haplotype diversity at the field level, we generated PacBio long-read amplicons for the known TSR genes *ACCase* and *ALS* and compared our empirical data with the results from probabilistic models of adaptation via selective sweeps and forward simulations. We infer that standing genetic variation is the most likely mechanism behind the TSR mutations of independent origin, with only a minor role for de novo mutations.

## Results

### Genome Assembly and Annotation.

**Fig. 1.**
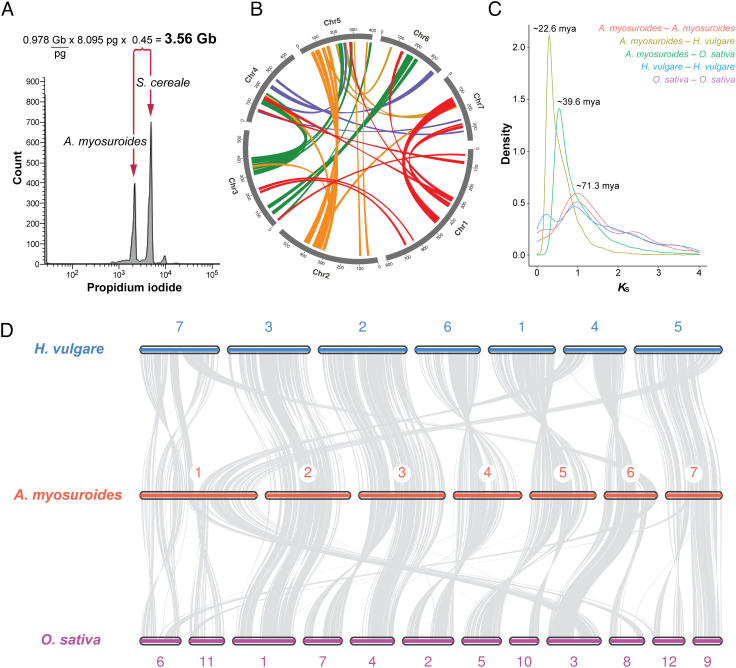
Reference genome of an *A. myosuroides* individual from a German herbicide-sensitive population. (*A*) Histogram of relative DNA content from flow cytometry of propidium iodide-stained nuclei of *A. myosuroides* and the reference standard *Secale cereale* cv. Daňkovské (diploid genome size = 16.9 pg). (*B*) Circos plot of the *A. myosuroides* genome, with colored lines connecting anchor pairs (genes in collinear regions) with synonymous substitution rates (*K*_S_) > 0.5. Numbers represent megabases. (*C*) *K*_S_ distributions for paralogs within the *A. myosuroides, Hordeum vulgare* (23), and *Oryza sativa* (24) genomes and for ortholog pairs shared by the three species. Divergence time, expressed as Mya, was estimated based on 7.0 × 10^−9^ as the substitution rate in grasses (25). (*D*) Syntenic relationships between the chromosomes of *A. myosuroides* and *H. vulgare* (*Top*) and *O. sativa* (*Bottom*).

For genome sequencing and assembly, we selected a single plant from an herbicide-sensitive population (Appels Wilde Samen GmbH, Darmstadt) from Germany and ascertained that it did not carry known TSR mutations at the nuclear *ACCase* and *ALS* or the chloroplast *psbA* loci (*Methods*). Previous genome size estimates of *A. myosuroides* based on Feulgen photometry ranged from 4.2 Gb (17) to 4.7 Gb (18). To estimate the genome size of the selected individual more accurately, we performed flow cytometry using rye (*Secale cereale*) as a reference standard (19), which yielded an estimated haploid genome size of 3.56 Gb (Fig. 1*A*). Next, we generated ~90× genome coverage of PacBio continuous long reads (CLRs), ~44× genome coverage of Illumina PCR-free short reads, and ~66× genome coverage of Hi-C chromatin contact data. We de novo assembled the genome with *FALCON-Unzip* (20), deduplicated primary contigs with *purge_dups* (21), and scaffolded contigs with *HiRise* (22) (Table 1). The size of the final assembly was 3.53 Gb and consisted of seven super-scaffolds (Fig. 1*B* and *SI Appendix*, Fig. S1*A*), in agreement with the known karyotype of the species with seven chromosomes (18).

**Table 1.** Genome assembly metrics

Descriptor	Value
Total assembly size	5,218,837,661 bp
Number of contigs	6,215
Contig N50	1,783,999 bp
Largest contig	11,174,859 bp
Chromosome-level assembly size	3,529,081,863 bp
Chromosome N50	554,019,051 bp
Largest chromosome	807,086,175 bp
Number of protein-coding genes	50,029
Mean gene length	2,789 bp
BUSCO score	C:94.6% [S:82.0%, D:12.6%], F:0.9%, M:4.5%
TE content	Class I [LTR: 63.8%, non-LTR: 0.1%] Class II [TIR: 10.9%, Helitron: 8.2%] Other repeated regions: 2.15%

Contig metrics are shown before deduplication. Benchmarking Universal Single-Copy Orthologs (*BUSCO*) (26) scores were obtained with the 'embryophyta_odb10' gene set (n = 1,614). Complete (C), single copy (S), duplicated (D), fragmented (F) and missing (M) genes are indicated. Transposable element (TE) content was determined with the *Extensive* de novo *TE Annotator* (EDTA) (27).

Given that in plants, transposable elements are a major driver of genome size, it is not surprising that repetitive sequences account for 85.2% of the *A. myosuroides* genome, with 63.8% classified as long terminal repeats retrotransposons (Table 1). We annotated 50,029 protein-coding genes based on a combination of both RNA-seq and PacBio Iso-Seq long transcripts from five different tissues (leaves, roots, whole inflorescences, anthers, and pollen), *ab initio* prediction, and protein homology. Transcriptome data supported 87.5% of the annotated genes, and 95% of all genes could be assigned functions with *InterProScan* (28). Of the 1,614 near-universal single-copy orthologs predicted by *BUSCO* (26), 94.6% were found as complete genes (82.0% as single copy, 12.6% as duplicated genes). On average, protein-coding genes in *A. myosuroides* are 2,789 bp long and contain 3.71 exons (Table 1). Potential shortcomings of our assembly are discussed in the *Methods* section.

Chromosome level synteny with other grasses was high, particularly with the more closely related *Hordeum vulgare* genome (23), for which the distribution of the number of synonymous substitutions per synonymous site (*K*_S_) for orthologous gene pairs indicated a divergence time of ~22.6 Mya (Fig. 1*C*). Chromosomes 2, 3, 4, 5, and 7 in *A. myosuroides* have a near 1:1 relationship with chromosomes 3, 2, 6, 1, and 5 in *H. vulgare*, respectively (Fig. 1*D*). An exception is chromosome 1 in *A. myosuroides* (807 Mb), which contains sequences that are syntenic with chromosomes 4, 5, and 7 in *H. vulgare*.

### Population Structure.

**Fig. 2.**
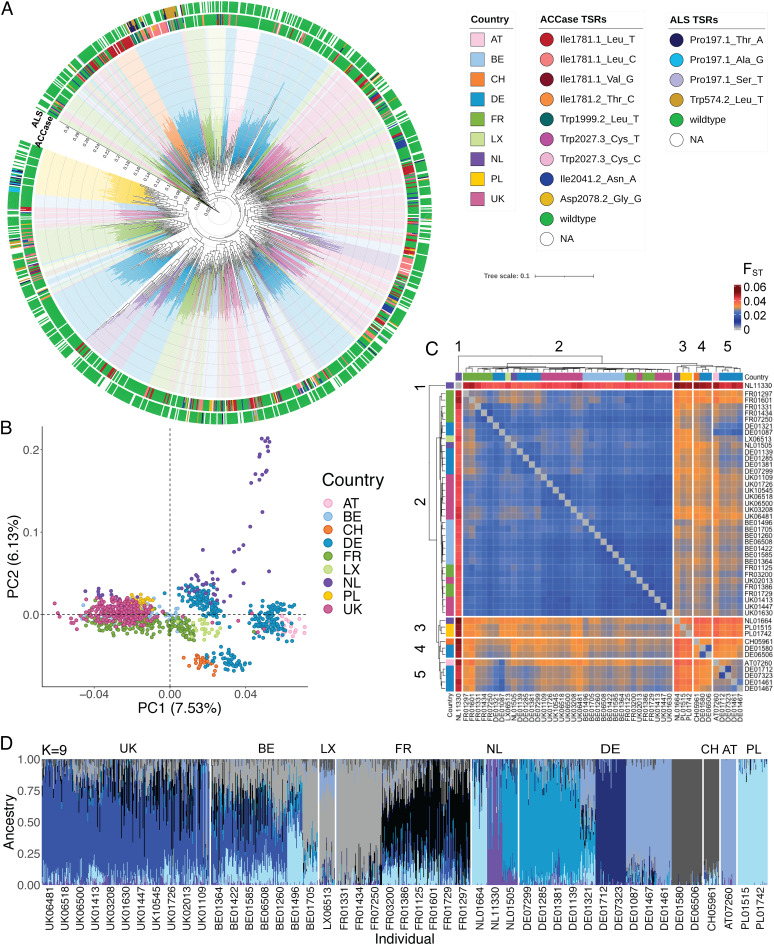
Population structure analysis of 47 European *A. myosuroides* populations with 109,924 genome-wide ddRAD-seq markers. (*A*) Maximum-likelihood tree. Branch ends are marked in country colors. TSR mutations for *ALS* and *ACCase* in each individual are indicated in the outer and inner rings, respectively. (*B*) Principal component analysis (PCA) showing the first two eigenvectors, with explained genetic variance in parentheses. Colors reflect country-specific origin of the populations. (*C*) Heatmap of fixation index (F_ST_) values in contrast between the different populations, ranging from close to 0 (blue) to 0.06 (dark red). Populations are clustered by similarity of F_ST_ patterns, and colors of the branch tips indicate countries of origin of the populations. (*D*) Admixture proportions with ancestry groups of K = 9. Admixture proportions with ancestry groups of K = 7 and K = 8 can be found in *SI Appendix*, Fig. S4. Each bar corresponds to one individual, grouped by country [Austria (AT), Belgium (BE), Switzerland (CH), Germany (DE), France (FR), Luxembourg (LX), Netherlands (NL), Poland (PL), United Kingdom (UK)].

Our new reference genome allowed us to easily assess the distribution of genome-wide diversity across Europe. To this end, we performed ddRAD-Seq in 1,123 individuals. These represented 44 populations, each with 22 to 24 plants, across nine European countries, and came from farmers with suspected herbicide resistance in their fields. For comparison, we included three herbicide-sensitive reference populations (Fig. 2). We defined 109,924 single nucleotide polymorphisms (SNPs) with an average sequencing depth of 22.6× (*SI Appendix*, Fig. S2*A*). A clear phylogeny per country was not discernible from the maximum likelihood (ML) tree (Fig. 2*A*), but Treemix captured the geographic distribution at the country scale without significant migration events, as indicated by the F3 statistic (*SI Appendix*, Fig. S3).

Overall genetic differentiation between populations was low (F_ST_ range: 0.01 to 0.05, n = 47; Fig. 2*C*), consistent with other studies of *A. myosuroides* (11, 12) and other wild grasses such as *Panicum virgatum* (29). The relatedness of individuals within populations was high (F_IS_ = 0.1; range 0.06 to 0.12). In the admixture analysis, we could identify between 7 and 9 ancestry groups (Fig. 2*D* and *SI Appendix*, Fig. S4*C*) that were consistent with the clusters formed in a principal component analysis (PCA; Fig. 2 *B* and *D* and *SI Appendix*, Fig. S4*A*). Individuals from Belgium (BE), the United Kingdom (UK), Luxemburg (LX), and France (FR) were genetically very similar and clustered together. A population from the Netherlands (NL), NL11330, was most differentiated from all others with F_ST_-values up to 0.05 (Fig. 2*C*). Germany (DE) was divided into three subclusters, one having common ancestry with individuals from Switzerland (CH), one with Austria (AT), and the third cluster being highly admixed. The populations from Poland (PL) shared common ancestry with the Netherland population NL01664. In summary, there is a clear geographical population structure across Europe, although the data at this point do not allow us to infer colonization and migration histories.

The mean observed SNP heterozygosity was 0.11, with no significant difference between populations that were under herbicide selection and those that were not (*SI Appendix*, Fig. S2*B*). We further estimated Watterson’s theta θ_W_ on the 1.1% sequenced fraction of our genome. The θ_W_ (mean = 0.0047) estimates are within the range of other outcrossing plant species (30). With these θ_W_ estimates and the mutation rate of 3.0 × 10^−8^ from maize (31), we determined effective population sizes ranging from 30,366 to 41,941 individuals (*SI Appendix*, Fig. S2*C*). Among countries for which we had more than six populations, Germany had significantly smaller (*P* value range: 0.01 to 0.03) effective population sizes than France, Belgium, and the United Kingdom (*SI Appendix*, Fig. S2*D*), but the causes for this difference remain unknown. Given that we have estimated effective population sizes in *A. myosuroides* mostly from populations under selection that have already experienced a decrease in population sizes, it is very likely that we rather underestimate the long-term effective population sizes of our *A. myosuroides* field populations (32). Messer and Petrov (2013) noted that temporal fluctuations in population size can strongly influence estimates of effective population size, especially in recent bottlenecks, as would be the case with adaptation processes to herbicides (33). Adaptation to strong selection pressure is a rapid process, and the probability of adaptive mutations arising is higher for larger population sizes (34, 35).

### Haplotype Networks of Herbicide Target Genes ALS and ACCase.

Much of the work on the molecular mechanisms underlying herbicide resistance has focused on mutations in the genes that encode the enzymes inhibited by herbicides. Two prominent herbicide targets are the genes encoding ALS and ACCase, both of which can be inhibited by a range of structurally diverse chemicals (36, 37). At both loci, mutations at multiple conserved codons are known to confer inhibitor resistance (4–7). To understand the diversity not only of specific mutations but also of entire haplotypes on which these mutations arose, we aimed to characterize the *ALS* and *ACCase* loci, including the extended linked sequences that surround them. We amplified ~13.2 kb for *ACCase* and ~3.6 kb for *ALS* by long-range PCR and analyzed complete amplicons with PacBio Circular Consensus Sequencing (CCS) for all individuals in our European collection. We applied very stringent criteria to call haplotype sequences in our dataset—requiring high accuracy (>99% or q20) and a minimal CCS read depth per sample of 25×. This enabled us to characterize entire haplotypes for 1,046 individuals for *ACCase* and 842 individuals for *ALS*. We were able to recover two haplotypes for the vast majority of our samples that passed quality control filters, 84.9% for *ACCase* and 59.8% for *ALS*. We assume that the remaining individuals are homozygous for the same haplotype.

**Fig. 3.**
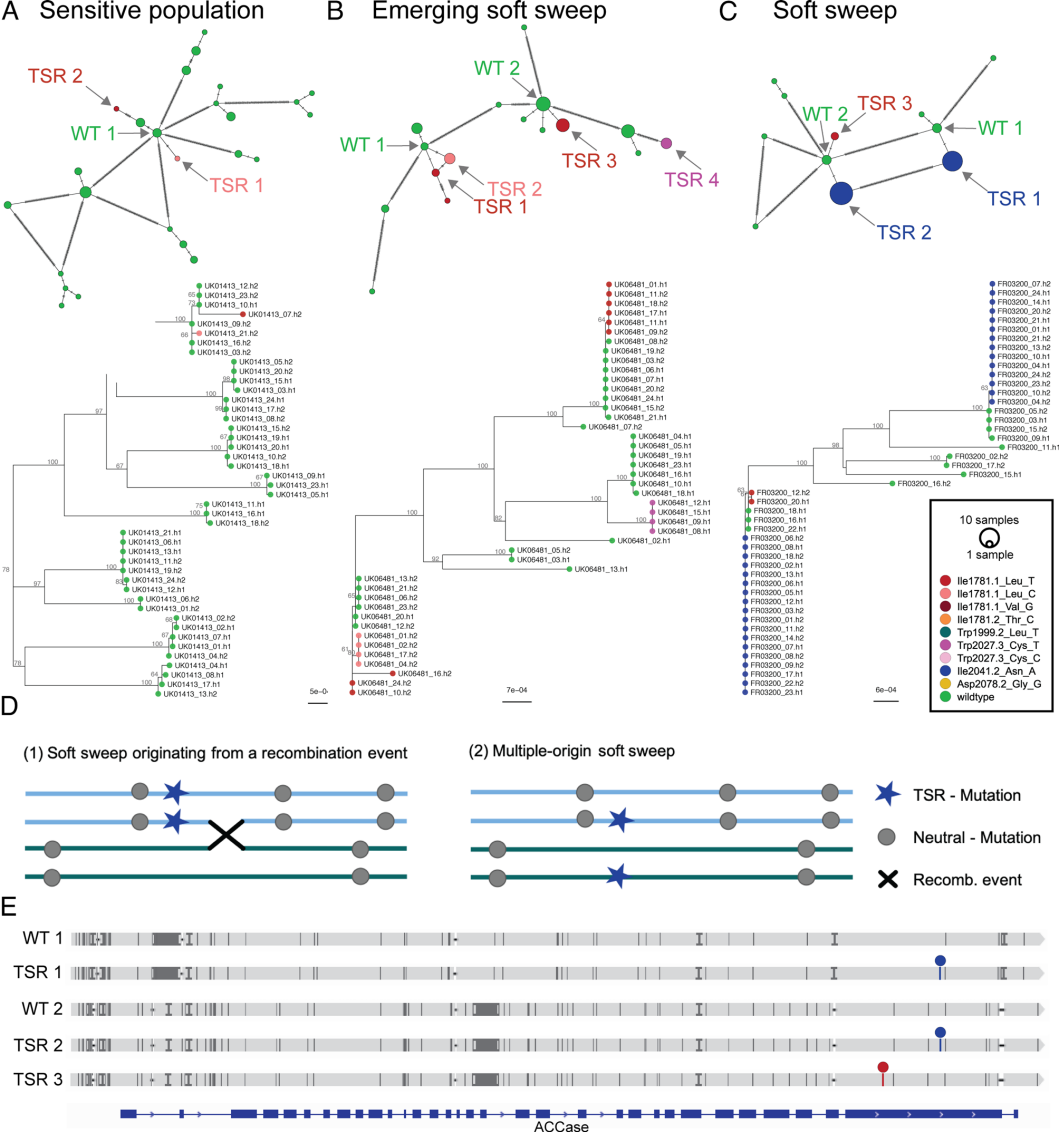
Haplotype analysis of the complete *ACCase* gene (13.2 kb). (*A*) Network and maximum likelihood (ML)-tree of 44 haplotypes from the sensitive reference population UK01413 (HerbiSeed standard), which has not been under herbicide selection. The color code in all networks and trees (*A*–*C*) indicates different target-site resistance (TSR) mutations, with haplotypes that have wild-type sequence at known TSR positions in green. Likely wild-type haplotypes of origin for TSR mutations are indicated (WT). (*B*) Network and ML-tree of 44 haplotypes from the British population UK06481, which shows a selection pattern characteristic of an emerging soft sweep for TSR mutations. (*C*) Network and ML-tree of 46 haplotypes from the French population FR03200, with a predominant soft sweep pattern for the TSR mutation Ile2041.2_Asn_A. (*D*) Schematic representation of alternative origins of soft sweep patterns: recombination vs. independent mutation events. (*E*) In the FR03200 population, two distinct wild-type haplotypes (WT 1 and WT 2) have independently sustained the same TSR mutation, giving rise to haplotypes TSR 1 and 2. In addition, wild-type haplotype WT 2 has given rise to a second TSR haplotype (TSR 3). Positions of TSR Ile1781.1_Leu_T and TSR Ile2041.2_Asn_A mutations are marked with red and blue circles, respectively.

Some TSR mutations were less common than others. For example, Trp2027Cys and Asp2078Gly in *ACCase* were underrepresented, consistent with these mutations reducing fitness in the absence of herbicide selection in other species (38–41). The most common mutation was Ile1781Leu, consistent with pleiotropic effects of this substitution that increase fitness also in the absence of selection (38, 39) (Fig. 3 *A*–*C* and Dataset S2). Finally, TSR mutations typically act in a dominant fashion, and the majority of TSR mutations (71.4%) at *ACCase* in our data occurred as heterozygotes.

PCA of *ACCase* haplotypes distinguishes three major groups (*SI Appendix*, Fig. S5). Although each group included representatives from all countries (including those countries for which we only analyzed single populations), and although alleles without obvious TSR mutations were present in all major groups, it is difficult to judge with current information whether the major groups of haplotypes arose before the species colonized Europe or whether they reflect relatively recent migration events. TSR substitutions Ile1781Leu, Ile1781Thr, Trp2027Cys, and Ile2041Asn were the most wide-spread and found in all European groups, while Asp2078Gly and Ile1781Val were found in two and Trp1999Leu in one group. This pattern of the same TSR mutation arising independently in separate geographic locations across Europe (Fig. 2*A*) extends previous observations made at local or country scales using small samples of short amplicons that included only a limited number of variable sites for haplotype detection (42–44).

To better characterize *ACCase* haplotype diversity, we inferred haplotype trees and networks at the level of single fields (Fig. 3 and Dataset S2). We observe haplotype networks of varying complexity (Fig. 3 *A*–*C*), likely reflecting the selection pressure to which each population was subjected. If the allele frequency of a single mutation—on a single haplotype—increases rapidly in a population, this is called a “hard sweep”. If, on the other hand, there are several different haplotypes in a population that confer resistance—whether they all carry the same beneficial mutation or different ones—and increase in frequency at the same time, this is referred to as a “soft sweep” (32). In our collection, only four out of the 27 populations with recorded TSRs contained a single TSR haplotype—and in these four cases, the TSR haplotypes were found at low frequency, with fewer than 10% of sequences having the corresponding TSR mutation. In principle, this pattern may well reflect an early state of a hard sweep, but that the other 23 populations contain at least two haplotypes with TSR mutations indicates that soft sweeps are the norm (*SI Appendix*, Table S1 and Fig. S6). In 14 of these populations, we found different haplotypes with the same TSR mutation resulting from multiple independent mutation events, as opposed to the same TSR mutation being transferred to other haplotypes by recombination (Fig. 3*D* and Dataset S2). This observation confirms in an unbiased manner inferences from earlier explorative studies (42–45). We found seven instances in which two or three different TSR mutations had arisen in a single field, from the same haplotype (Fig. 3 *B*, *C*, and *E* and Dataset S2). The maximum number of independent (nonrecombinant) *ACCase* TSR haplotypes within a field population was 10 (*SI Appendix*, Table S1).

Having observed multiple TSR haplotypes in the same field, we also asked the converse question, whether the same haplotypes could be found across populations. The *ACCase* sequences from our collection of 1,046 individuals could be clustered into 250 nonredundant haplotypes, of which a quarter (n = 62) carried one of the known TSR substitutions (Dataset S1). A third of these (n = 20) were shared across multiple populations, which was essentially the same as wild-type haplotypes shared by multiple populations (55 out of 188; chi-squared test *P* value = 0.7736). Furthermore, the most common TSR haplotypes had evolved from the most common wild-type haplotypes. For instance, three of the eight most abundant TSR haplotypes were only one mutation away from the single most abundant wild-type haplotype, which made up 12.6% of all individual haplotypes (n = 264). We note that identical TSR haplotypes do not have to have a single origin, given that parallel herbicide resistance evolution is common, and identical TSR mutations can occur not only on different *ACCase* haplotypes in *A. myosuroides* but also in other genes associated with resistance to herbicides of different species (46).

In the complete assembly of the *A. myosuroides* genome, we discovered at least two copies of the *ALS* gene in chromosome 1 (*Methods*). These copies have full-length open reading frames without introns, and high-quality Iso-Seq reads (>99.9% or q30) span full-length transcripts (*SI Appendix*, Fig. S7). The copy most similar to the GenBank sequence AJ437300.2 (47) was designated as *ALS1*, and we selectively amplified *ALS1* with primers that should not target the other *ALS* loci (or locus). The existence of multiple *ALS* copies in *A. myosuroides* may have confounded previous studies, which relied on primers in the coding region to genotype *ALS* TSR mutations. This strategy was used in a pyrosequencing assay (48) commonly used for this type of study, which, differently from our work, did not lead to the identification of homozygous or trans-heterozygous Pro197Thr genotypes (49, 50).

*ALS1* haplotypes fell into three major Europe-wide groups (*SI Appendix*, Fig. S8). In our collection, TSR mutations for this gene were only present in Germany, France, United Kingdom, and Poland. TSR mutations Pro197Thr and Trp574Leu were found in two of these groups, Pro197Ala and Pro197Ser only in one, and no obvious TSR mutation was found in the third group. Similar to *ACCase*, although less often, two or more TSR haplotypes of independent origin could be detected within single fields, in six of the nine populations with recorded TSRs (*SI Appendix*, Table S1 and Dataset S3).

### Simulations of Standing Genetic Variation vs. de novo Mutations.

Strong selection pressure exerted by herbicides leads to very rapid adaptation, but a major question is whether herbicide resistance evolves predominantly from standing genetic variation that was present already before the onset of herbicide selection or from de novo mutations that arose after herbicide selection began. In other words, are the typical population dynamics in terms of effective population size and drift compatible with a reservoir of TSR mutations available before exposure to herbicides and are spontaneous TSR mutations sufficiently frequent for rapid resistance evolution?

To answer this question, we first used equations from Hermisson and Pennings (34) to derive expectations for the probability of adaptation (i.e., evolution of herbicide resistance via TSR mutations) and the likelihood that this adaptation is due to standing genetic variation. First, we calculated the probability of adaptation, assuming a mutation rate of 3.0 × 10^−8^ (31), a mutational target size of seven nucleotides, corresponding to the TSR mutations investigated here, and onset of herbicide selection 30 generations ago. As mentioned above, estimates of N_e_ based on genetic diversity integrate over a long period of time and past bottlenecks will reduce it, leading to estimates that are lower than the actual N_e_ before the bottlenecks (33). Therefore, we considered both N_e_ = 42,000, which is the highest estimate from our field populations, and N_e_ = 84,000.

**Fig. 4.**
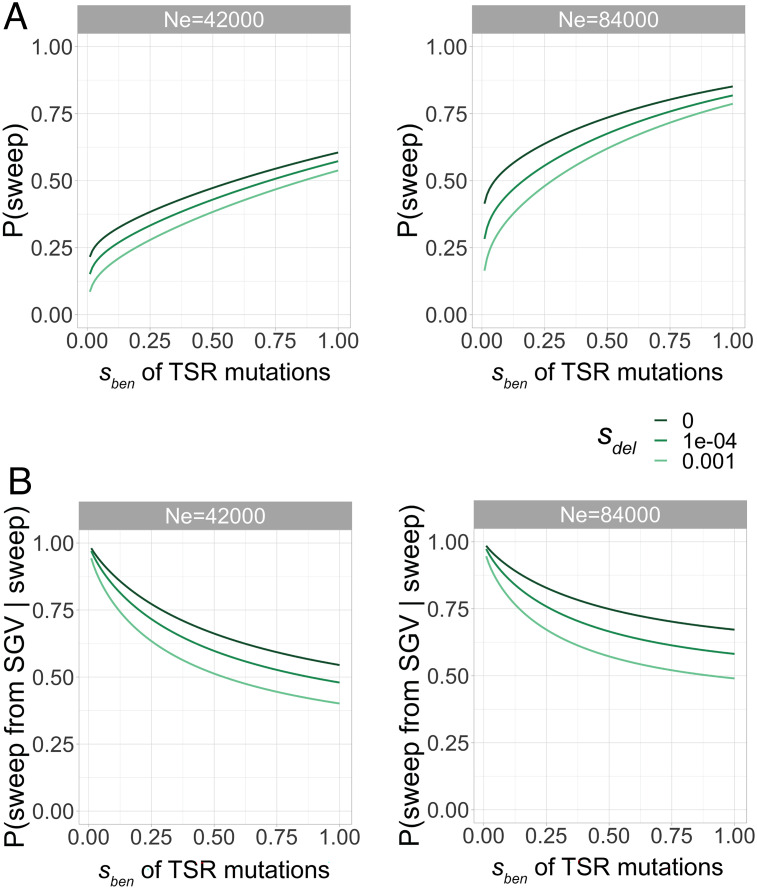
Simulations of different scenarios for adaptation. Equations from Hermisson and Pennings (34) were used to derive (*A*) expectations for the probability of adaptation via a selective sweep from beneficial target-site resistance (TSR) mutations in general and (*B*) from standing genetic variation in particular. Probabilities of sweeps with different effective population sizes (N_e_) are estimated as a function of the strength of selection.

With N_e_ = 42,000, we observed that the probability of adaptation strongly depends on the beneficial selection coefficient of the TSR mutation during the herbicide selection phase (*s_ben_*). For strong positive selection (*s_ben_* ≥ 1), which we expect for herbicide application, the probability of adaptation is high (> 50%). Only for weakly beneficial mutations (*s_ben_* ≤ 0.01) it decreases below 20% (Fig. 4 *A*, *Left*). With N_e_ = 84,000, the probability of adaptation increases to up to 80% due to both higher levels of standing genetic variation and a larger rate of de novo mutations (Fig. 4 *A*, *Right*). The deleterious selection coefficient of TSR mutations before the onset of herbicide selection has only a minor influence on the probability of adaptation.

Next, we asked whether adaptation to herbicide selection pressure predominantly occurs via standing genetic variation or de novo mutation (34). We observed that fixation from standing genetic variation is more probable (>50%) for neutral or almost neutral mutations (*s_del_* < 1e^−4^) and has a probability larger than 40% even for deleterious mutations (*s_del_* = 1e^−3^) and the smaller population size (N_e_ = 42,000) (Fig. 4 *B*, *Left*). The probability of adaptation from standing genetic variation generally increases with smaller *s_ben_* or larger *N_e_* (N_e_ = 84,000; Fig. 4 *B*, *Right*) because of the decreasing fixation probability of de novo mutations and the increasing levels of standing genetic variation, respectively (34). These results suggest that herbicide resistance should occur predominantly, although not exclusively, via standing genetic variation.

**Fig. 5.**
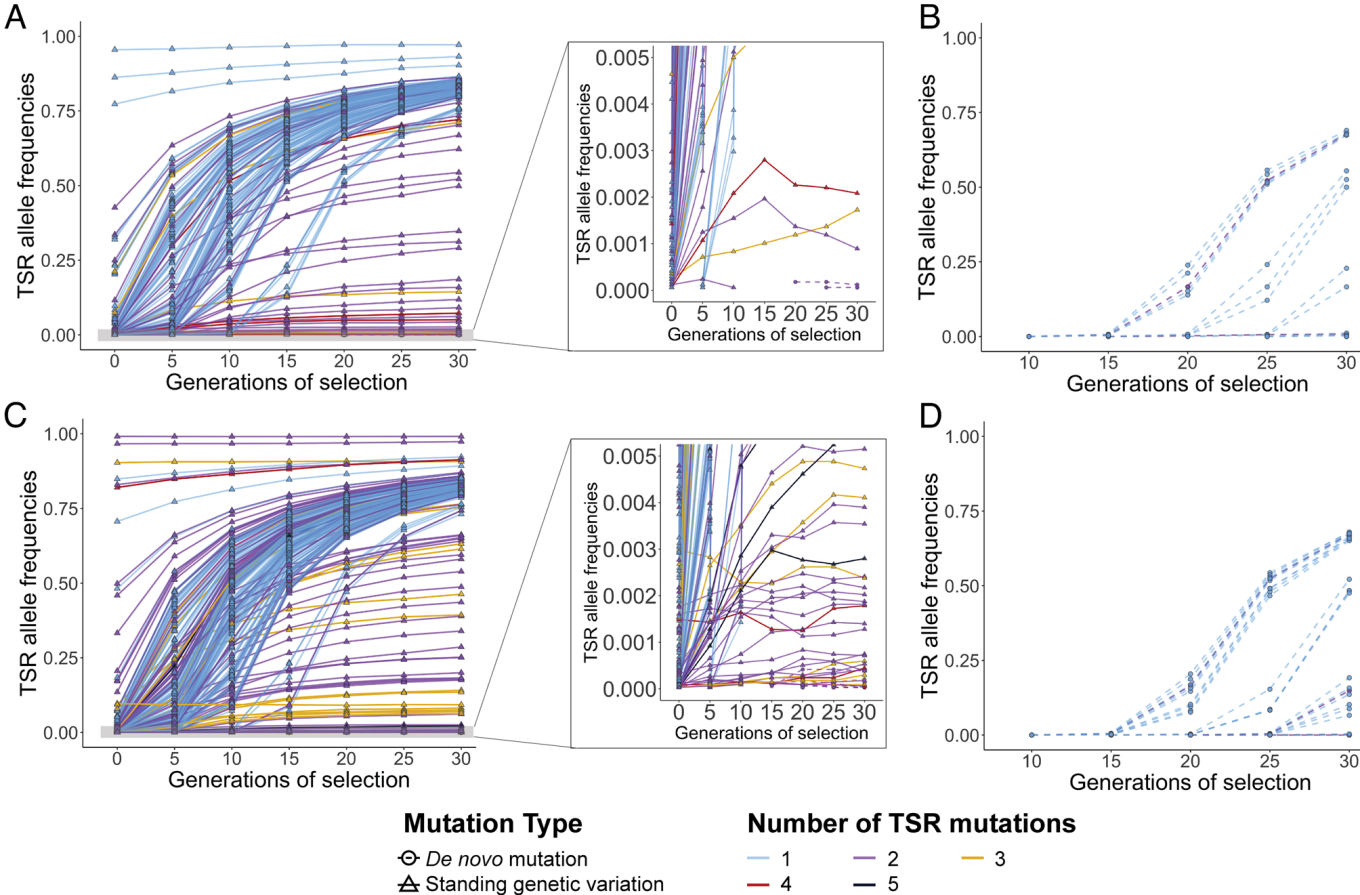
Simulations of expected allele frequencies for TSR alleles arising from standing genetic variation or de novo mutation. The distribution of mutations was generated with a generic gene model that has the same number of exons and introns, and ratio between coding and noncoding sequences as the *ACCase* gene. Mutations in introns and noncoding regions were considered to be neutral, while exons had a ratio of 0.25/0.75 (neutral/deleterious) mutations according to Messer and Petrov (52), with selection coefficients (s) for deleterious mutations drawn from a gamma distribution with E[s] = −0.000154 and a shape parameter of 0.245 (51). Five hundred of one thousand simulation runs are shown for an effective population size (N_e_) of (*A* and *B*) 42,000 individuals and (*C* and *D*) 84,000 individuals. Continuous lines represent mutations originating from standing genetic variation; de novo TSR mutations are shown with dashed lines. Colors indicate the total number of TSR mutations per population. (*A* and *C*) Standing genetic variation scenario, with TSR mutations preexisting in the populations before herbicide selection. Shown is the increase in TSR allele frequencies under herbicide selection of up to 30 generations, with one herbicide application per generation. The right panel shows a truncated *Y* axis at 0.005 TSR allele frequencies. Notice that some TSR de novo mutations have also arisen in runs that had preexisting TSR alleles. (*B* and *D*) De novo mutation scenario. Any TSR mutation that might have arisen before the start of selection has been lost again, so that no TSR mutations are present at generation 0 of selection.

The remarkable diversity of TSR haplotypes of independent origin observed in individual fields (Fig. 3) prompted us to examine the speed of adaptation and the expected level of TSR diversity in more detail through forward-in-time simulations with the software SLiM (16). We defined two possible scenarios in which TSR mutations arise: one in which resistant alleles were already present in the population before the start of herbicide selection (standing genetic variation) and one in which they emerged only after selection pressure was imposed (de novo mutation) (*SI Appendix*, Fig. S9). Our model assumes that individuals with at least one TSR mutation have a 20 times higher chance of surviving the herbicide treatment than individuals without any TSR mutation. We applied a gamma distribution of deleterious mutations in exons (51) (Fig. 5), but a model with exclusively neutral mutations gave similar results (*SI Appendix*, Fig. S10). We ran one thousand simulations for two different N_e_ values, analyzing the changes in allele frequencies of TSR mutations, and the number of independent TSR mutations per population.

With N_e_ = 42,000, TSR mutations were more often attributable in the simulations to standing genetic variation (25.5%) than solely de novo mutations (4.2%). Also, we found more independent TSR mutations per population for the standing genetic variation scenario, at least two mutations in 41 simulation runs, with a maximum of four mutations in one run (Fig. 5*A*). Under the de novo scenario, there were at most two mutations, which were found in a single run (Fig. 5*B*). Since we had observed in most of our populations with TSR, namely in 23 out of 27, at least two independent TSR mutations, the simulations tend to agree mostly with standing genetic variation as a main driver of herbicide resistance evolution in our system. However, even under this scenario, the simulations fell short with respect to the extent of TSR due to soft sweeps observed in our populations.

Highly effective population sizes favor rapid adaptation processes because advantageous alleles are more likely to be immediately available and at higher frequencies (32, 34). With N_e_ = 84,000, 40.3% simulation runs had TSR mutations due to standing genetic variation and 5.7% had de novo mutations (Fig. 5 *C* and *D*), favoring the former scenario even more than with N_e_ = 42,000. Also, we found up to five independent TSR haplotypes due to standing genetic variation (Fig. 5*C*). In the single simulation run—under the model that considered deleterious mutations in exons—that led to five independent TSR mutations, these were already present at the onset of selection.

Even with a larger N_e_, our simulations seem to underestimate both the ratio of TSR soft sweeps over hard sweeps, as well as the maximum number of independent TSR haplotypes in a given population, indicating that our N_e_ estimates are conservative. Actual N_e_ might be even higher since estimates based on RAD-seq data tend to underestimate genetic diversity (53, 54), and empirical census population sizes are higher than our estimated N_e_ (55). Very high effective population sizes would be consistent with reports from farmers of heavy infestations in fields due to difficulties in managing resistance. Large effective population sizes likely reflect large census population sizes, thus maintaining genetic variation under herbicide selection and providing a large genetic pool for accumulation of resistance mutations. Factors that promote high census population sizes in specific years include climatic variables that lead to poor weed control conditions, seed dormancy, reduced tillage efficiency, large seed banks, and crop rotations with high amounts of winter cereals (1, 55, 56).

Simulations have shown that in the time it takes for a particular allele to become fixed in a population starting from standing genetic variation, the same mutation can arise de novo (34). In the case of de novo mutations, our simulations reveal that there is a considerable risk that they are directly lost again through drift since they are on average initially much rarer than mutations that are part of standing genetic variation (Fig. 5 *A* and *C* and *SI Appendix*, Fig. S10 *A* and *C*), added to the possibility that some TSR mutations are slightly beneficial even in the absence of herbicide application (38, 39). And although we cannot exclude de novo mutations as a source of TSR alleles, they are characterized by a slow initial phase of adaptation (after 10 to 15 generations under selection) in our simulations, thus cannot compete with preexisting mutations from standing genetic variation (Fig. 5 *B* and *D* and *SI Appendix*, Fig. S10 *B* and *D*). Therefore, the standing genetic variation scenario, with the presence of multiple alleles, as is typical for soft sweeps, is closer to what we observed in our experimental data. Furthermore, to estimate how many TSR alleles per generation are present as standing genetic variation in a field, we ran 100 simulations under neutrality. This revealed the emergence and loss due to random genetic drift in field populations before the start of herbicide selection by farmers. We could detect up to four TSR alleles at the same time (*SI Appendix*, Fig. S11).

Previous studies documented rapid adaptation of grass weeds to herbicide applications within a few generations, sometimes as quickly as three or four generations (57, 58), which is in agreement with anecdotal reports from farmers. The degree of herbicide resistance in a field is also closely correlated with the frequency of application (59). This would be consistent with TSR mutations having been present already at low frequency before herbicides came into use, as shown through herbicide treatment of naive populations of *Lolium rigidum* (60) or the analysis of herbarium samples of *A. myosuroides* collected before the advent of modern herbicides (61). In the case of *L. rigidum*, the frequency of sulfometuron-methyl resistance in previously untreated populations was around 10^−4^ (60) while among 685 *A. myosuroides* herbarium specimens, one individual collected nearly hundred years before the introduction of herbicides carried the *ACCase* Ile-1781-Leu mutation (61). In fact, we found TSR mutations in two of our three sensitive reference populations by deep amplicon sequencing (HerbiSeed standard, WHBM72 greenhouse standard APR/HA from September 2014), although it is unknown when these populations were collected with respect to the relevant herbicides coming into broad use. On the other hand, in a study that aimed to empirically determine the de novo mutation rate of TSRs in a natural population of grain amaranth (*Amaranthus hypochondriacus*), not a single spontaneous resistant genotype was found among 70 million screened plants (62). This would give 1.4 × 10^−8^ as an approximate upper bound of spontaneous mutations conferring resistance to a specific herbicide, which is in the range of spontaneous mutation rates that have been empirically measured in plants for single sites (63, 64).

## Discussion

Plants have evolved a remarkable number of mechanisms to protect themselves against damage and extinction from changing environmental conditions, including ones due to human activity. In particular, the outsized selection imposed by repeated application of herbicides had led to extraordinarily rapid evolutionary adaptation in many weed species.

While several TSR mutations incur fitness penalties in the absence of herbicide applications (40, 41), some do not, and there is even at least one report of a TSR mutation being favorable independently of herbicide application (38, 39). The extent of fitness costs before herbicide application began in a population will in turn affect whether TSR mutations can accumulate in a population that is not under herbicide selection. Our study shows that standing genetic variation is primarily responsible for the observed level of per-field diversity of TSR haplotypes of independent origin that is associated with the rapid evolution of herbicide resistance in *A. myosuroides* populations. This suggests that the TSR mutations that are the focus of our investigation have limited fitness costs in the absence of herbicide treatment.

Another factor that likely influences the speed of TSR resistance is the presence and abundance of NTSR alleles, which in turn will be affected by the herbicide regime in that particular population. For example, herbicide mixtures promote unspecific resistance through NTSR due to enhanced metabolism of herbicides with diverse modes of action (65). In our collection, there is a substantial fraction of individuals resistant to either ACCase or ALS inhibitors that cannot be attributed to known TSR mutations (*SI Appendix*, Fig. S12). The ratio of TSR to NTSR varies greatly, with some populations having only one or the other, and other populations having both (*SI Appendix*, Fig. S13). In many cases, evolutionary adaptation in response to a change in the environment occurs via soft selective sweeps, as this allows a greater proportion of ancestral genetic diversity to be maintained (66). In our case, there was a wide range in the fraction of TSR individuals per population but no correlation with genome-wide nucleotide diversity (π) (Pearson’s r: 0.26, *P*-value: 0.075) (*SI Appendix*, Fig. S14), consistent with previous analyses using AFLP markers (11). The preservation of genetic diversity is particularly important in agricultural fields with highly variable conditions in terms of crop rotation, pest management, and other field management measures and can be crucial for weed populations to thrive under a range of different environmental conditions. Resistance apparently evolves in parallel in numerous fields with weed populations, occurring through different mechanisms that nature has at its disposal—TSR at a particular locus being one of them—depending on which resistance pathways have preexisting mutations.

Highly accurate long-reads have enabled us to resolve entire haplotypes of TSR genes and thus to ascertain their independent origin. To determine the contribution of gene flow, the reconstruction of larger haplotypes extending dozens or hundreds of kilobases will be necessary. Haplotype information can be inferred from whole-genome shotgun sequencing data by ancestral recombination graphs (67) or by targeted long-read sequencing to reassemble a larger genomic region. The high-quality genome assembly we have disclosed here provides a foundation for such future analyses.

In conclusion, our examination of different scenarios for adaptation to herbicides indicates that with the diversity of resistance mechanisms available, a large fraction of *A. myosuroides* populations is likely to have the genetic prerequisites not only for rapid evolution of resistance to currently used herbicide modes of action but also to potential new future modes of action.

## Materials and Methods

For detailed experimental and analytical procedures, please see *SI Appendix*, *Supporting Text*.

### Reference Genome.

A single plant from an herbicide-sensitive population (Appels Wilde Samen GmbH, Darmstadt) from Germany was sequenced with CLR in a PacBio Sequel I system. FALCON-Unzip toolkit was used for initial assembly (20), and contigs were subjected to deduplication with purge_dups v1.0.0 (21). Hi-C library reads were used as input data for HiRise for chromosome-level scaffolding (22). To aid gene annotation, both Illumina RNA-seq and PacBio Iso-seq data from five tissues (anthers, whole inflorescences, leaves, pollen, and roots) from the same individual were generated.

### Population Studies.

For the population structure analysis of 47 European *A. myosuroides* populations, the ddRAD libraries were prepared according to a published method for fresh samples (68) and sequenced in an Illumina NovaSeq 6000 system on a S2 FlowCell in paired-end mode and with a read length of 150 bp to an average coverage of 22.6x read depth. Variants were called with GATK v4.1.3.0 (69), and SNPs were filtered following the recommendations of the RAD-Seq variant-calling pipeline ‘dDocent’ (70). The ML phylogenetic tree that shows the genetic relationship between the samples of our European dataset was inferred with RAXML-NG v0.9.0 (71) and visualized with the interactive Tree Of Life online tool (72) (Fig. 2*A*). The identification of ancestry groups was performed with ADMIXTURE (73) (Fig. 2*D*). Effective population sizes were calculated after the formula N_e_ = θ_W_/4*μ for a diploid organism. Watterson thetas θ_W_ were estimated with ANGSD v0.930 (74) exclusively from the ddRAD-sequenced portion of the *A. myosuroides* assembly. The mutation rate *μ* = 3.0 × 10^−8^ was adopted from *Zea mays* (31).

### Amplicon Analysis.

*ALS* and *ACCase* long-range amplicons were generated with the barcoded primers listed in Dataset S1, sequenced in a PacBio Sequel I system, and converted to haplotypes with the tool PacBio Amplicon Analysis (https://github.com/PacificBiosciences/pbAA). Multiple alignments of all haplotypes per population were performed with MAFFT v7.407 (75), trees were inferred with RAXML-NG v0.9.0 (71), and minimum spanning networks were visualized with POPART v.1.7 (76) (Fig. 3 and Datasets S2 and S3).

### Simulations.

To model the general probability of adaptation through a sweep and then specifically from standing genetic variation (Fig. 4), we used equations 8, 11, 14, 18, and 20 from Hermisson and Pennings (34). For forward-in-time simulations (Fig. 5 and *SI Appendix*, Fig. S10), we used the software SLiM v3.4 (16) with the *ACCase* locus (12,250 bp) as a template, a burn-in period of 10 × *N_e_* generations, and 30 generations of selection (*SI Appendix*, Fig. S9). Both the mutation rate (3.0 × 10^−8^) (31) and genome-wide average recombination rate (7.4 × 10^−9^) (77) were adopted from *Z. mays*. We set the population size to 42,000 individuals, which is the highest possible *N_e_* from the populations characterized with RAD-Seq data. Since diversity estimates of *N_e_* integrate over a long period of time and past bottlenecks will reduce it, leading to estimates that are lower than the actual *N_e_* before the bottlenecks (33), we additionally simulated the doubled effective population size of 84,000 individuals.

## Supplementary Material

Appendix 01 (PDF)

## Data Availability

Raw data including PacBio CLR and Iso-seq reads, Illumina PCR-free, Hi-C, and RNA-seq reads can be accessed in the European Nucleotide Archive (ENA; https://www.ebi.ac.uk/ena/browser/home) under project accession number PRJEB49257 (78), assembly accession CASDCE010000000 (79). Raw ddRAD-seq data for the population study, and PacBio CCS q20 reads can be downloaded from the ENA project accession number PRJEB49288 (80). Annotation files for the genome assembly, the SNP matrix for the ddRAD-seq experiment, and the fasta files with the haplotypes of *ACCase* and *ALS* can be found at https://doi.org/10.5281/zenodo.7634530 (81). Scripts and experimental protocols to reproduce the analyses in this study are deposited in the GitHub repository of this study (https://github.com/SonjaKersten/Herbicide_resistance_evolution_in_blackgrass_2022) (82).
