# Supplementary material for: Standing genetic variation fuels rapid evolution of herbicide resistance in blackgrass
Source: Proc Natl Acad Sci U S A. 2023 Apr 12;120(16):e2206808120. doi: 10.1073/pnas.2206808120 (PMC10120058; doi:10.1073/pnas.2206808120)
Supplement: Supplementary file 1 — Appendix 01 (PDF) [file pnas.2206808120.sapp.pdf]

PNAS

Supporting Information for

Standing genetic variation fuels rapid evolution of herbicide resistance in blackgrass

Sonja Kersten[1,2], Jiyang Chang[3,4], Christian D. Huber[5], Yoav Voichek[6], Christa Lanz[2], Timo Hagmaier[2], Patricia Lang[2†], Ulrich Lutz[2], Insa Hirschberg[7], Jens Lerchl[8], Aimone Porri[8], Yves Van de Peer[3,4,9,10], Karl Schmid[1], Detlef Weigel[2], and Fernando A. Rabanal[2]

[1]Institute of Plant Breeding, Seed Science and Population Genetics, University of Hohenheim, 70599 Stuttgart, Germany.
[2]Department of Molecular Biology, Max Planck Institute for Biology Tübingen, 72076 Tübingen, Germany.
[3]Department of Plant Biotechnology and Bioinformatics, Ghent University, 9052 Ghent, Belgium.
[4]Center for Plant Systems Biology, Vlaams Instituut voor Biotechnologie, 9052 Ghent, Belgium.
[5]Department of Biology, The Eberly College of Science, Penn State University, State College, PA 16801, USA.
[6]Gregor Mendel Institute, Austrian Academy of Sciences, Vienna BioCenter, 1030 Vienna, Austria.
[7]Friedrich Miescher Laboratory, 72076 Tübingen, Germany.
[8]Agricultural Research Station, BASF SE, 67117 Limburgerhof, Germany.
[9]Department of Biochemistry, Genetics and Microbiology, University of Pretoria, Pretoria 0028, South Africa.
[10]College of Horticulture, Academy for Advanced Interdisciplinary Studies, Nanjing Agricultural University, Nanjing, 210095, China.
[†]current address: Department of Biology, Stanford University, Stanford, CA 94305, USA.

Detlef Weigel
Email: weigel@weigelworld.org

This PDF file includes:

Supporting text
Figures S1 to S14
Tables S1 and S2
Legends for Datasets S1 to S3
SI References

Other supporting materials for this manuscript include the following:

Datasets S1 to S3

Supporting Information Text

Reference genome sequencing, assembly and annotation

Plant selection and flow cytometry

A single plant from a sensitive German reference population provided by BASF was selected. All required tissues for all described reference related sequencing methods were collected from the same plant. We confirmed the absence of known TSR mutations on the *ACCase*, *ALS* and *psbA* loci using Illumina amplicon sequencing. PCR products of the three target genes (Dataset S1) were pooled, and sequencing libraries were generated with a purified *Tn5* transposase as described in a previous study (1). The library was spiked into an Illumina HiSeq 3000 lane. The resulting reads were checked for known TSR mutations causing herbicide resistance (2, 3).

Leaf tissue from both the selected *A. myosuroides* plant and the reference standard *Secale cereale* cv. Daňkovské (4) were simultaneously chopped with a razor blade in 250 µl of nuclei extraction buffer (CyStain PI Absolute P kit; P/N 05-5022). After the addition of 1 ml of staining solution (including 6 µl of propidium iodide (PI) and 3 µl of RNase from the same kit) the suspension was filtered through a 30 µm filter (CellTrics®; P/N 04-0042-2316). Five replicates of these samples were stored in darkness for 4 h at 4°C prior to flow cytometry analysis. PI-area was detected with a BD FACSMelody™ Cell Sorter (BD Biosciences) equipped with a yellow-green laser (561 nm) and 613/18BP filtering. A total of 25,000 events were recorded per replicate, and the ratio of the mean PI-area values of each target sample and reference standard 2C peaks was used to estimate DNA content according to ref. (5) (mean = 3.53 Gb; s.d. = 0.0052 Gb; n = 5).

Whole-genome PacBio sequencing

Prior to high-molecular weight (HMW) extraction the reference plant was kept for 48 hours in the dark to reduce the starch accumulation. We harvested ca. 30 g of young leaf material and ground it in liquid nitrogen. Nuclei isolation was performed according to a published protocol (6) with the following modifications: we used 16 reactions, each with 1 g input material in a 20 ml nuclear isolation buffer. The filtered cellular homogenate was

centrifuged at 3500 x g, followed by 3x washes in nuclear isolation buffer. The isolated plant cell nuclei were resuspended in 60 µl Proteinase K (#19131, Qiagen). For HMW-DNA recovery, the Nanobind Plant Nuclei Big DNA Kit (SKU NB-900-801-01, Circulomics) was used. In total, we obtained approximately 80 µg of HMW-DNA, which was subjected to needle shearing once (FINE-JECT® 26Gx1" 0.45x25mm, LOT 14-13651). A 75-kb template library was prepared with the SMRTbell® Express Template Preparation Kit 2.0, and size-selected with the BluePippin system (SageScience) with 15-kb cutoff and a 0.75% agarose, 1-50kb cassette (BLF7510, Biozym) according to the manufacturer's instructions (P/N 101-693-800-01, Pacific Biosciences, California, USA). The library was sequenced on a Sequel I system (Pacific Biosciences) using the Binding Kit 3.0. and MagBead loading. In total, we sequenced 18 SMRT cells of 10 hours and 8 SMRT cells of 20 hours movie time.

Illumina PCR-free library sequencing

The genomic DNA was fragmented to 350 bp size using a Covaris S2 Focused Ultrasonicator (Covaris) with the following settings: duty cycle 10%, intensity 5, 200 cycles and 45s treatment time. The library prep was performed according to the manufacturer's instructions for the NxSeq® AmpFREE Low DNA Library Kit from Lucigen® (Cat No. 14000-2) with the addition of a large-cutoff bead-cleanup (0.6 : 1, bead:library ratio) after the adapter ligation, followed by the recommended standard bead-cleanup at the final purification step. The library was quantified with the Qubit Fluorometer (Invitrogen) and quality checked on a Bioanalyzer High Sensitivity Chip on an Agilent Bioanalyzer 2100 (Kit #5067-4626, Agilent Technologies). The library was sequenced on two lanes of an Illumina HiSeq 3000 system in paired-end mode and with a read length of 150bp.

Short-read RNA-seq

RNA was extracted from five tissues (leaves, whole inflorescences, anthers, pollen, roots) following a published protocol (7). Remaining DNA was removed with DNaseI (#EN0521, Thermo Scientific) following manufacturer's recommendations. The quality was checked with an RNA 6000 Nano Chip on an Agilent Bioanalyzer 2100 (Kit

#5067-1511, Agilent Technologies). All RNA integrity number (RIN) scores were above 5.4.

For the library preparation, the NEBNext Ultra II Directional RNA Library Prep Kit for Illumina in combination with the Poly(A) mRNA Magnetic Isolation Module (#E7760, #E7490, NEB) was used. The heat fragmentation was performed for a duration of 9 min resulting in final library sizes of around 545 bp. All 5 libraries were equally pooled and sequenced on one lane of an Illumina HiSeq 3000 system in paired-end mode and with a read length of 150 bp.

Long-read PacBio RNA Iso-seq

We extracted RNA from the same five tissue samples as for the short-read sequencing. To ensure a high RNA quality for long-read sequencing, we used a published protocol (8), which is a CTAB based method for high-quality total RNA applications from different plant tissues. The remaining DNA was removed with the TURBO DNA-free Kit (Invitrogen), designed for optimal preservation of RNA during the DNase treatment. The quality check on the Agilent Bioanalyzer 2100 (Agilent Technologies) with an RNA Nano 6000 Chip resulted in RIN scores higher than 7.6 for all tissues.

The IsoSeq libraries were prepared following the PacBio protocol for 'Iso-Seq™ Express Template Preparation for Sequel and Sequel II Systems' (P/N 101-763-800 Version 02; October 2019, Pacific Biosciences, California, USA). The cDNA was amplified in 12 cycles and purified using the 'standard' workflow for samples primarily composed of transcripts centered ~2 kb.

Hi-C library preparation

Hi-C libraries were prepared in a similar manner as described (9). Briefly, for each library, chromatin was fixed in place with formaldehyde in the nucleus and then extracted. Fixed chromatin was digested with DpnII, the 5' overhangs filled in with biotinylated nucleotides, and then free blunt ends were ligated. After ligation, crosslinks were reversed, and the DNA purified from protein. Purified DNA was treated to remove biotin that was not internal to ligated fragments. The DNA was then sheared to ~350 bp

mean fragment size and sequencing libraries were generated using NEBNextUltra enzymes and Illumina-compatible adapters. Biotin-containing fragments were isolated using streptavidin beads before PCR enrichment of each library. The libraries were sequenced on an Illumina HiSeq X.

Genome assembly

Genome assembly was done with the FALCON and FALCON-Unzip toolkit (10) distributed with the 'PacBio Assembly Tool Suite' (falcon-kit 1.3.0; pypeflow 2.2.0; https://github.com/PacificBiosciences/pb-assembly). For the pre-assembly step, in which CLR subreads are aligned to each other for error correction, we opted for auto-calculating our own seed read length ('length_cutoff = -1') with 'genome_size = 3530000000' and 'seed_coverage = 40' . Details of the FALCON assembly parameters used in this study are provided in the dedicated GitHub for this study (https://github.com/SonjaKersten/Herbicide_resistance_evolution_in_blackgrass_2022). Primary contigs were subjected to deduplication with purge_dups v1.0.0 (11) using cutoffs (5, 36, 60, 72, 120, 216). For scaffolding, deduplicated primary contigs and Hi-C library reads were used as input data for HiRise, a software pipeline designed specifically for using proximity ligation data to scaffold genome assemblies (12). Dovetail Hi-C library sequences were aligned to the draft input assembly using bwa (13). The separations of Dovetail Hi-C pairs mapped within draft scaffolds were analyzed by HiRise to produce a likelihood model for genomic distance between read pairs, and the model was used to identify and break putative misjoins, to score prospective joins, and make joins above a threshold.

Genome annotation

Transposable elements were annotated with the tool Extensive *de-novo* TE Annotator (EDTA) v1.9.7 (14). The protein-coding gene annotation pipeline involved merging three independent approaches: RNA-aided annotation, *ab initio* prediction and protein homology search. The first approach is based on both RNA-seq and Iso-seq data from five tissues, anthers, whole inflorescences, leaves, pollen and roots. Pre-processing of Iso-seq data was carried out with PacBio® tools (https://github.com/PacificBiosciences/pbbioconda) that included in a first step the

generation of Circular Consensus Sequencing (CCS) reads (minimum predicted accuracy 0.99 or q20) with ccs v5.0.0 and demultiplexing with lima v2.0.0. In a second step, poly-A trimming and concatemer removal were done at the sample level (i.e., separately for each tissue) while clustering was carried out for all tissues combined with functions from isoseq3 v3.4.0. Unique isoforms had a mean length of 2,210 bp.

Iso-seq clusters were aligned to the *A. myosuroides* genome using GMAP v2017-11-15 using default parameters(15), whereas RNA-seq datasets were first mapped to the *A. myosuroides* genome using Hisat2 (16) and subsequently assembled into transcripts by StringTie2 (17). All transcripts from Iso-seq and RNA-seq were combined using Cuffcompare (18). Transdecoder v5.0.2 (https://github.com/TransDecoder) was then used to find potential open reading frames (ORFs) and to predict protein sequences. To further maximize sensitivity for capturing ORFs that may have functional significance, BLASTP(19) (v2.6.0+, arguments -max_target_seqs 1 -evalue 1e-5) was used to compare potential protein sequences with the Uniprot database (20). In the second approach, *ab initio* prediction was performed by BRAKER2 (21) using a model trained with RNA-seq data from *A. myosuroides*. For the third approach, consisting of homology prediction, the protein sequences from five closely related species (*Brachypodium distachyon*, *Oryza sativa*, *Setaria italica*, *Sorghum bicolor* and *Hordeum vulgare*) that belong to the same family were used as query sequences to search the reference genome using TBLASTN (e < 1e-5). These databases were downloaded from Plaza v4.5 (22) (https://bioinformatics.psb.ugent.be/plaza/). Regions mapped by these query sequences were subjected to Exonerate (23) to generate putative transcripts.

Finally, EvidenceModeler v1.1.1 (24) was used to integrate all of the above sources of evidence, and the Benchmarking Universal Single-Copy Orthologs (BUSCO; v4.0.4; embryophyta_odb10) gene set to assess the quality of annotation results (25). Putative gene functions were identified using InterProScan (26) with different databases, including PFAM, Gene3D, PANTHER, CDD, SUPERFAMILY, ProSite, GO. Meanwhile, functional annotation of these predicted genes was obtained by aligning the protein sequences of these genes against the sequences in public protein databases and the UniProt database using BLASTP (e-value $<1 \times 10^{-5}$).

Comparative genomics

Analyses related to synonymous substitution rates (K_S) were performed using the wgd package (27). First, the paranome (entire collection of duplicated genes) was obtained with 'wgd mcl' using all-against-all BLASTP and MCL clustering. Then, the K_S distribution of *A. myosuroides* was calculated using 'wgd ksd' with default settings, MAFFT V7.453 (28) for multiple sequence alignment, and codeml from PAML package v4.4c (29) for maximum likelihood estimation of pairwise synonymous distances. Anchors or anchor pairs (duplicates located in collinear or syntenic regions of the genome) were obtained using i-ADHoRe (30), employing the default settings in 'wgd syn'.

Plant genomes typically contain both whole-genome and segmental duplications. We therefore investigated collinear regions indicative of recent duplications. When we analyzed the divergence of closely related paralogs present in these regions based on synonymous substitution rates (K_S), we noticed two main peaks, one at K_S ~0.16 and another one at K_S ~1.2 (Figure S1B). The K_S of the first peak is unusually low and would normally indicate very recent duplicates. To explore the nature of the gene pairs with low K_S, we extracted all gene pairs in these regions with $K_S \leq 0.5$ and asked how they are distributed in the genome. Collinear blocks containing these pairs are generally very close and always within the same chromosome (Figure S1C), while pairs with $K_S > 0.5$ are located in different chromosomes (Figure 1B). One explanation would be that these blocks are the products of recent duplication events, although there is not much evidence for large-scale local duplications in plant genomes. Alternatively, they could be an artifact of the assembly process, as in highly heterozygous genomes, different alleles can be assembled independently into different contigs. If these duplicates are not properly purged, which is particularly difficult if alleles are very dissimilar, then during scaffolding they are placed close to each other on the same chromosome. With the data at hand, it is difficult to distinguish between these two possibilities, but based on the close paralogs being almost always present close to each other, we favor the second explanation. The second peak (K_S ~1.2), mostly representing paralogs in different chromosomes (Figure 1B, Figure S1B), coincides with a known whole-genome duplication (WGD) event common in all grasses (31, 32) that occurred ~70 million years ago (mya). The list of anchors and their K_S values is available in Dataset S1.

MCscan JCVI (33) was used to do the analysis of syntenic relationships and depth ratio by providing the coding DNA sequences (CDS) and annotation file in gff3 format. TBtools was used to visualize the results via a Circos plot (34).

Population studies

Sample collection and DNA extraction

Seeds from 44 *A. myosuroides* populations from nine European countries were provided by BASF. The seeds were collected from farmers with suspected herbicide resistance in their fields against ACCase – and/or ALS-inhibiting herbicides. In addition, we included three sensitive reference populations (HerbiSeed standard, Broadbalk long-term experiment Rothamsted 2013, WHBM72 greenhouse standard APR/HA from September 2014).

The seeds of all 47 populations were sown in vermiculite substrate and stratified in a 4°C climatic chamber for one week, and subsequently placed in the greenhouse at 23°C / 8 h daytime, 18°C / 16 h nighttime regime. After one week in the greenhouse, one plant per pot was transferred to standard substrate (Pikiererde Typ CL P, Cat. No. EN12580, Einheitserde) for a total of 27 plants per population. We aimed to collect 8-weeks-old leaf tissue from 24 individuals per population, but due to insufficient germination in two populations, we were unable to collect material from two individuals and therefore finally obtained 1,126 samples for further processing. 300 mg of plant material was collected into a 2 ml screw cap tube filled with 4-5 porcelain beads and ground with a FastPrep tissue disruptor (MP Biomedicals). For DNA extraction, we used a lysis buffer consisting of 100 mM Tris (pH 8.0), 50 mM EDTA (pH 8.0), 500 mM NaCl, 1,3% SDS and 0.01 mg/ml RNase A. The DNA was precipitated with 5M potassium acetate, followed by two bead-cleanups for DNA purification. For a detailed hands-on protocol, see https://github.com/SonjaKersten/Herbicide_resistance_evolution_in_blackgrass_2022.

Phenotyping

For phenotyping, 27 plants per population described in the previous section were divided into two treatment and two control groups, following a specific tray design to minimize

spatial growth effects. Treatment 1: Atlantis WG® (Bayer Crop Science) + Synergist Atlantis WG® (10 plants per population). Control 1: Only Synergist Atlantis WG® (three plants per population). Treatment 2: Axial® 50 (Syngenta) + Synergist Hasten (10 plants per population). Control 2: Only Synergist Hasten (four plants per population). All plants were sprayed 11 weeks after transplanting. Herbicides and synergists were applied with a lab sprayer (Schachtner), nozzle Teejet 8001 EVS and an air pressure of between 200-225 kPa. The sprayer was calibrated for a field application rate of 400 l/ha in four rounds of three independent replicates each (M = 396.4, SD = 7.53). Axial® 50 (50 g/l of pinoxaden + 12.5 g/l Cloquintocet-mexyl) was applied in combination with the synergist Hasten (716 g/l rapeseed oil ethyl and methyl esters, 179 g/l nonionic surfactants, ADAMA Deutschland GmbH). Atlantis WG® (29.2 g/kg of mesosulfuron and 5.6 g/kg of iodosulfuron) was used with the provided synergist (276,5 g/l sodium salt, fatty alcohol ether sulfate, Bayer Crop Science). Control plants were sprayed only with the synergists. Axial® 50 was applied at the recommended field rate of 1.2 l/ha , Atlantis WG® at 800 g/ha and both synergists at 1 l/ha. After four weeks all plants were scored according to the scheme in (Figure S12A), where the score D1 represents completely dead plants and the score A6 represents plants without any growth reductions compared to the control plants of the respective population.

ddRAD library preparation and sequencing

The ddRAD libraries were prepared according to a published method for fresh samples (35). 200 ng input DNA per sample were digested with the two restriction enzymes EcoRI (#FD0274, Thermo Fisher Scientific) and Mph1103I (FD0734, Thermo Fisher Scientific), followed by double-stranded custom-adapter ligation. The custom-adapters contain different numbers of additional nucleotides to shift the sequencing of the restriction enzyme sites and prevent the sequencer from causing an error due to unique signaling. After the restriction enzyme digestion step and the adapter ligation, large cutoff bead-cleanups (0.6:1, bead:library ratio) with homemade magnetic beads (Sera-Mag SpeedBeads™, #65152105050450, GE Healthcare Life Sciences) in PEG/NaCl buffer (36) were used to clean the samples from the buffers and remove large fragments above ~600 bp length. We used a dual-indexing PCR to be able to multiplex up to six 96-well plates of samples. Thus, two pools of libraries were sufficient for all our samples. Since it is challenging to determine exact library concentrations, our strategy

consisted of pooling all samples to the best of our abilities with the concentrations at hand and spike them into an Illumina HiSeq 3000 lane for about 5% of the total coverage. Afterwards, the library concentrations were re-calculated from the read coverage output and re-pooled accordingly to achieve a more even coverage. Size selection was performed using a BluePippin system (SageScience) with a 1.5% agarose cassette, 250bp-1.5kb (#BDF1510, Biozym) for a size range of 300–500 bp. The library pools were quantified with the Qubit Fluorometer (Invitrogen) and quality checked on a Bioanalyzer High Sensitivity Chip on an Agilent Bioanalyzer 2100 (Kit #5067-4626, Agilent Technologies). A detailed hands-on protocol can be found here: https://github.com/SonjaKersten/Herbicide_resistance_evolution_in_blackgrass_2022.

First, each library pool was sequenced in-house on an Illumina HiSeq 3000 lane in paired-end mode and 150 bp read length to assess the performance and quality. Afterwards, both pools were submitted to CeGaT GmbH, Tübingen, and sequenced with an Illumina NovaSeq 6000 system on a S2 FlowCell with XP Lane Loading in paired-end mode and with a read length of 150 bp. Total data output was 1.4 Tb, representing an average coverage of 22.6x read depth.

Alignment, SNP calling and SNP filtering

Demultiplexed raw reads were first trimmed for the base-shifts of the custom adapters in the 5' and 3' fragment ends. Afterwards, all remaining adapter sequences and low-quality bases were removed and only reads with a minimum read length of 75 bp were kept using cutadapt v2.4 (37). The read quality was checked before and after trimming with FastQC v0.11.5 (http://www.bioinformatics.babraham.ac.uk/projects/fastqc). Paired-end reads were first merged using Flash v1.2.11 (38), then the extended and the unmerged reads were independently aligned to the reference genome using bwa-mem v0.7.17-r1194-dirty (13). We used samtools v1.9 (39) to sort and index the bam-files and to finally combine the bam files of the extended and unmerged aligned reads per sample.

Variant calling was performed with the HaplotypeCaller function of GATK v4.1.3.0 (40). For joint genotyping, we broke the reference at N-stretches and generated an interval list with Picard's v2.2.1 function 'ScatterIntervalsByNs'

(http://broadinstitute.github.io/picard/). Next, we generated a genomic database by using GATK v4.1.3.0 'GenomicsDBImport', followed by joint genotyping with 'GenotypeGVCFs'. A first missing data filter (--max-missing 0.3) was applied with VCFtools v0.1.15 (41) to the VCF outputs of all intervals to reduce the number of unusable variants. Afterwards all interval VCFs were merged with Picard v2.2.1 'MergeVcfs'. The combined VCF was filtered following the recommendations of the RAD-Seq variant-calling pipeline ‘dDocent’ (42). First, basic filters were applied with VCFtools v0.1.15 (--max-missing 0.5 --mac 3 --minQ 30 --minDP 3 --max-meanDP 35), followed by advanced filter options for RAD-Seq data with 'vcffilter' (ABHet > 0.25 & ABHet < 0.75 | ABHet < 0.01 & QD > 5 & MQ > 40 & MQRankSum > (0 - 5) & MQRankSum < 5 & ExcessHet < 30 & BaseQRankSum > (0 - 5) & BaseQRankSum < 5) (https://github.com/vcflib/vcflib). We also filtered individuals with missing data more than 0.5, which removed four individuals from our dataset, and we ended up with a total of 1,122 individuals. Lastly, we used a population specific variant filter, which allowed for 30% missing data, but every variant had to be called in at least 10 populations. Our final VCF for further analysis contained 109,924 informative SNPs.

Phylogeny and population genetics statistics

A maximum likelihood (ML) phylogenetic tree was inferred with RAXML-NG v0.9.0 (43) to display the genetic relationship between the samples of our European dataset. We inferred a single ML-tree without bootstrapping using the model GTR+G+ASC_LEWIS of nucleotide evolution with ascertainment bias correction since we inferred it on RAD-seq data. The annotation of the tree for the known TSR mutations was done based on the *ALS* and *ACCase* amplicons described below. For visualization, we used the interactive Tree Of Life (iTOL) online tool (44).

To assess the population structure of our European collection we ran a principal component analysis (PCA) with the R-package SNPrelate (45) on 101,114 biallelic informative SNPs. To perform the admixture analysis on shared ancestry, we first pruned the dataset with PLINK v1.90b4.1 (46) for only biallelic SNPs. Admixture v1.3.0 (47) was run for up to 10 k groups, using a 10-fold cross-validation procedure to infer the right amount of k groups. TreeMix v1-13 (48) was run on the VCF filtered with PLINK as previously described in the admixture analysis. The transformation into the right input file

format was done with STACKS v1.48 (--treemix) (49). The tree was rooted with the most divergent outgroup population NL11330 (-root NL11330) and inferred in windows of 50 SNPs (-k 50) with 5 bootstrap replicates (-bootstrap 5). Since the treemix F3 statistic did not show significant migration, no migration events were added to the tree. FSTs were calculated with STACKS v1.48 (--fstats) (49) and visualized with the R package ComplexHeatmap 2.0.0 (50).

Since we only covered about 1.1% of the entire genome with our ddRAD-Seq reads, we calculated the Watterson thetas θ_W and effective population sizes exclusively from the sequenced portion of our genome. Therefore, we used ANGSD v0.930 (51) on our previously generated bam-files and applied some basic filters (-uniqueOnly 1 -remove_bads 1 -only_proper_pairs 0 -trim 0 -C 50 -baq 1 -minMapQ 20), followed by calculation of the site-frequency spectra (SFS) (-doCounts 1 -GL 1 -doSaf 1) and Watterson's theta estimator θ_W in sliding windows of 50,000 bp with a step size of 10,000 bp. The effective population size was calculated after the formula $N_e = \theta_W / 4*\mu$ for a diploid organism. The mutation rate μ for the calculation was taken from the *Zea mays* literature (52) as a genome-wide average of 3.0×10^{-8}.

VCFtools v0.1.15 (41) was used to calculate the coverage (--depth) of the SNP markers and the observed homozygosity O(HOM) (--het). Using the number of sites N_SITES, the proportion of observed heterozygous sites can be calculated according to the formula (N_SITES - O(HOM)) / N_SITES.

ALS **and** ***ACCase*** **amplicon analysis**

ALS and *ACCase* PacBio amplicon sequencing

To generate *ALS* and *ACCase* amplicons for long-read PacBio sequencing we used the same DNA from the European collection described in a preceding section. Before PCR amplification DNA was normalized to 10 ng/µl. Then, 30 ng (*ALS*) and 50 ng (*ACCase*) total input DNA was used for the PCR Master Mix reaction (1 µl P5 indexing primer (5 µM), 1 µl P7 indexing primer (5 µM), 4 µl of 5x Prime STAR buffer, 1.6 µl dNTPs, 0.4 µl Prime STAR polymerase (Takara, R050B), filled up to 20 µl with water). The indexing PCR program for *ALS* was a 2-step PCR with 10 seconds of denaturation at 98°C and 210 seconds of annealing and extension at 68°C for 28 cycles, followed by a final

extension for 10 min at 72°C. For *ACCase*, the annealing and extension step was elongated to 660 seconds. Amplicons were then pooled equally per gene and bead cleaned. In the case of the 13.2 kb amplicon from *ACCase*, we added a BluePippin (SageScience) size selection to remove any remaining fragments below 10 kb. PacBio libraries were created according to the following PacBio amplicon protocol (part number 101-791-800 version 02 (April 2020)) and SMRT cells were loaded on a PacBio Sequel I system with Binding Kit and Internal Ctrl Kit 3.0 (part number 101-461-600 version 10; October 2019). An extended hands-on protocol can be found at https://github.com/SonjaKersten/Herbicide_resistance_evolution_in_blackgrass_2022.

PacBio amplicon analysis

Most steps were carried out with tools developed by PacBio (https://github.com/PacificBiosciences/pbbioconda). First, CCS reads were generated with ccs v6.0.0 (minimum predicted accuracy 0.99 or q20). Then, demultiplexing was carried out with lima v1.11.0 (with parameters '--ccs --different --peek-guess --guess 80 --min-ref-span 0.875 --min-scoring-regions 2 --min-length 13000 --max-input-length 14000' for *ACCase* while for *ALS* similar parameters were used except for '--min-length 3200 --max-input-length 4200'). Next, pbaa cluster (v1.0.0) was run with default parameters followed by a series of amplicon-specific filtering steps.

For *ACCase*, we required a minimum of 25 CCS reads per sample, and only "passed clusters" were further considered for analysis. Samples with either 0 or more than 2 clusters were discarded. In samples in which a single cluster was identified (i.e., homozygous individuals for this locus), both haplotypes were assigned the same cluster sequence. In samples in which two different clusters (haplotypes) were identified, the difference between their respective frequencies had to be ≤ 0.50, otherwise the sample was discarded.

In the case of *ALS*, PCR amplification had been uneven, as our primers preferentially amplified certain haplotypes in individuals heterozygous for this locus. We presumed this was due to various structural variations downstream of the gene between major haplotypes (Figure S7). Therefore, to be able to analyze haplotype diversity of this locus, we employed less strict filtering steps than for *ACCase*. For *ALS*, a minimum of 25 CCS

reads per sample were required, while both 'passed clusters' and originally 'failed clusters' (mostly due to low frequency) were re-evaluated. First, only samples with cluster diversity ≤ 0.40 and cluster quality ≥ 0.7 were kept. In samples in which a single cluster was identified (i.e., homozygous individuals for this locus), its frequency had to be ≥ 0.98 to then assign the same cluster sequence to both haplotypes. In samples in which two different clusters (haplotypes) were identified, the difference between their respective frequencies was allowed to be ≤ 0.85, otherwise the sample was discarded. In the few samples in which three or more different clusters (haplotypes) were identified, the sum of the frequencies of the two main clusters had to be ≥ 0.96, and their difference ≤ 0.85 to be considered for downstream analyses.

Haplotype networks, haplotype trees and haplotype PCA

To annotate the clusters generated with pbaa with TSR metadata information, the single cluster fasta files representing two alleles per individual were first converted to fastq files using 'Fasta_to_fastq' (https://github.com/ekg/fasta-to-fastq). The resulting fastq files were aligned to the *ACCase* reference using minimap2 v2.15-r913-dirty (53), followed by sorting and indexing of the output bam files with samtools v1.9 (39). Read groups were assigned with the Picard function 'AddOrReplaceReadGroups' (RGID=$SAMPLE RGLB=ccs RGPL=pacbio RGPU=unit1 RGSM=$SAMPLE) (http://broadinstitute.github.io/picard/), followed by variant calling using GATK v4.1.3.0 (40) with functions 'HaplotypeCaller' (-R $REF --min-pruning 0 -ERC GVCF) and 'GenotypeGVCFs' with default settings. Variant annotation in the resulting VCF was performed with SnpEff v4.3t (54). The VCF was loaded in R to extract the TSR information and annotate the haplotype networks, trees and PCA with custom R scripts.

For the multiple alignments per population, we first combined all respective individual fasta files of the pbaa clusters into a single fasta file and then aligned them using MAFFT v7.407 (--thread 20 --threadtb 10 --threadit 10 --reorder --maxiterate 1000 --retree 1 --genafpair) (28). We used PGDSpider v2.1.1.5 (55) to transfer the multiple alignment fasta file into a Nexus-formatted file. Minimum spanning networks were inferred and visualized with POPART v.1.7 (56). Per population haplotype trees were inferred with RAXML-NG v0.9.0 (43) from the multiple sequence alignment files. 'Tree search' was performed with 20 distinct starting trees and bootstrapping analysis with the model

GTR+G and 10,000 bootstrap replicates. Tree visualization was done in R with ggtree v1.16.6 (57). The packages treeio v1.8.2 (58) and tibble v3.0.4 (https://github.com/tidyverse/tibble/) were used to add the TSR metadata information to the tree object. The branch length and node support values were extracted from Felsenstein's bootstrap proportions (FBP) output files. The haplotype PCAs were performed using the R package SNPrelate (45) on the previously generated VCFs for ALS and ACCase and visualized using ggplot2 (59).

Identification of *ALS* copies

Using the *ALS* GenBank sequence of *A. myosuroides* AJ437300.2 (60) as a query, BLASTN v2.2.29+ (61) retrieved three hits in chromosome 1 of our assembly. These loci corresponded to three gene models annotated as the largest subunit of ALS: model.Chr1.12329 (identity = 1921/1923 bp; 99.8%; hereafter *ALS1*), model.Chr1.11275 (identity = 1820/1915 bp; 95.0%; hereafter *ALS2*) and model.Chr1.11288 (identity = 1818/1915; 94.9%; hereafter *ALS3*).

To better characterize the relationship between these putative copies of the *ALS* gene, we analyzed synonymous substitution rates (K_S) and Iso-seq full-transcripts. K_S values between paralogs *ALS1*-*ALS2* and paralogs *ALS1*-*ALS3* were 0.153 and 0.165, respectively, while between paralogs *ALS2*-*ALS3* was 0.028. Although all K_S values between these paralogs were below 0.5, they are not present in our list of anchor pairs from the comparative genomics analysis (Dataset S1) for not being located among the collinear regions identified by i-ADHoRe (30).

For the analysis of Iso-seq data, we first generated very high-quality reads, with a minimum predicted accuracy 0.999 or q30, per tissue up until the poly-A trimming and concatemer removal step with isoseq3 v3.4.0 as described before for genome annotation. Next, we combined the q30 Iso-seq transcripts from all tissues and extracted only those that matched the following internal *ALS* sequences conserved among the three loci: 'CGCGCTACCTGCCCGCCTC', 'GTCTCCGCGCTCGCCGATGCT, 'GTCCAAGATTGTGCACAT' and 'GAGTGAAGTCCGTGCAGCAATC'. We obtained 343 Iso-seq q30 full-length transcripts, and it is worth mentioning that different internal *ALS* sequences yield near identical numbers of transcripts. Since Iso-seq q30 reads have

heterogeneous lengths, we used cutadapt v2.4 (37) to trim all reads at the 5' and 3' borders (-a CTTATTAATCA -g CCACAGCCGTCGC) of the CDS to make them all the same length. Finally, clustering with pbaa v1.0.0 (--min-read-qv 30) resulted in only three clusters with 143 reads corresponding to *ALS1*, 100 reads to *ALS2* and 100 reads to *ALS3*. Representative full-length Iso-seq reads with average read quality of q93 from each cluster were used for Figure S7. Therefore, all *ALS* gene models can produce full-length transcripts. Taking together K_S values and Iso-seq data, we could only conclude that *ALS1* is clearly distinct from *ALS2* and *ALS3*, but we could not distinguish whether *ALS2* and *ALS3* are two distinct loci or two alleles of the same locus.

Model simulations

Using equations 8, 11, 14, 18 and 20 from Hermisson and Pennings (62), we first modeled the general probability of adaptation through a sweep and then specifically from standing genetic variation. We set the population size to 42,000 individuals, which is the highest possible N_e from the populations characterized with RAD-Seq data. Since diversity estimates of N_e integrate over a long period of time and past bottlenecks will reduce it, leading to estimates that are lower than the actual N_e before the bottlenecks (63), we additionally simulated the doubled effective population size of 84,000 individuals. As maize is a diploid grass with a similar genome size to *A. myosuroides*, we adopted the mutation rate 3.0 x 10^{-8} (52). Both target site resistance genes in our study contain seven well described SNP positions that cause resistance (2, 3, 64, 65), therefore we set the mutational target size to seven. Before selection, we assumed three different selection coefficients for those mutations: 0, 1e-04, 0.001. Under selection, those TSR positions were beneficial in a range from 0 to 1 (Figure 4A,B, x-axes). The number of generations of selection was set to 30.

Standing genetic variation model vs. *de novo* model

Forward simulations were executed on a computing cluster with SLiM v3.4 (66) using SLiMGui v3.4 for model development. We used the *ACCase* locus (12,250 bp) as a template for all our simulations. Since we sequenced 585 bp upstream and 364 bp downstream of the gene, we defined the length of our simulated genomic element as 13,199 bp with TSR mutations at the following positions: 11052 (Ile1781), 11706

(Trp1999), 11790 (Trp2027), 11832 (Ile2041), 11943 (Asp2078), 11973 (Cys2088), 11997 (Gly2096). We further defined three genomic element types: exon, intron and non-coding region. For introns and non-coding regions, all mutations were considered to be neutral. In exons, a ratio of 0.25/0.75 (neutral/deleterious) mutations was used according to Messer and Petrov (67), with selection coefficients (s) for deleterious mutations drawn from a gamma distribution with E[s] = -0.000154 and a shape parameter of 0.245 (68). Since *A. myosuroides* is an annual grass, all models were built as Wright-Fisher models with non-overlapping generations and standard Wright-Fisher model assumptions (http://benhaller.com/slim/SLiM_Manual.pdf, p.35/36). As described above, we set the population size to 42,000 and 84,000 individuals. Both the mutation rate (3.0×10^{-8}) (52) and genome-wide average recombination rate (7.4×10^{-9}) (69) were adopted from maize. We implemented a burn-in period of 10 x N_e generations to generate the initial genetic diversity and, since this is a computationally intensive process, we scaled our models down by a factor of 5.

We ran the model in one thousand independent runs per population size (42,000 and 84,000 individuals), and with (Figure 5) or without exons and introns, in which case all mutations were considered to be neutral (Figure S10), until generation 10 x N_e. After this generation, we applied herbicide selection for which mutations at the specified TSR positions became highly beneficial and dominant, with a selection coefficient s_i of 1.0 and a dominance coefficient h_i of 1.0 (fitness model for TSR individuals, homozygous: $1 + s_i = 1 + 1 = 2$, and heterozygous: $1 + h_i * s_i = 1 + 1 * 1 = 2$) (Figure S9). In practice, an herbicide is usually applied in the field once or twice each year. Since in *A. myosuroides* one generation time corresponds to about one year, we simulated one selection event per generation. Foster *et al.* 1993 (70)) specifically reported an ACCase inhibiting herbicide efficiency rate of 95-97%. However, it is likely that some *A. myosuroides* plants without TSR mutations will later emerge and thus escape the lethal effect of herbicide treatment contributing to the genetic diversity in the field. Therefore, in our simulations, we assume a remaining fitness of 10% for individuals that do not carry a TSR mutation to account for plants that escaped herbicide treatment or germinated at a later time point (fitness model for individuals without TSR, homozygous: $1 + s_i = 1 + (-0.9) = 0.1$). The selection pressure was applied at the end of every generation for a total of 30 generations. Only survivor individuals could reproduce and contribute to the next generation.

Generation 10 x N_e was a checkpoint for the presence of TSR mutations. If at least one individual in the total population was carrying at least one of the TSR mutations in a heterozygous state, the run belonged to the standing genetic variation scenario. If the first TSR mutation emerged only after herbicide selection, the run belonged to the *de novo* scenario. TSR allele frequencies and proportion of resistant individuals for 7 different time points (before selection, 5, 10, 15, 20, 25 and 30 generations after start of selection) were written to a log file and plotted with ggplot2 (59).

TSR occurrence

Furthermore, we examined how often a TSR mutation occurs on our simulated *ACCase* locus and how long it remains in the population before either being lost due to genetic drift or increase in frequency toward fixation under neutral conditions. This allows us to quantify how often resistance mutations are present as standing genetic variation in a field population before herbicide selection starts. To this end, we used a modified version of the model described in the previous section, this time without preexisting mutations, and ran it for 1,000 generations under neutrality. The other general parameters stayed the same as described above: 42,000 and 84,000 individuals, maize mutation rate 3.0 x 10^{-8} [(52)], maize recombination rate 7.4 x 10^{-9} [(69)]. Mutations were modeled using the described intron/exon gene model for the *ACCase* locus. After each generation, we output the number of TSR mutations at the predetermined TSR positions in the population. We performed 100 independent simulation runs per N_e. Detailed scripts for all simulations can be found at https://github.com/SonjaKersten/Herbicide_resistance_evolution_in_blackgrass_2022.

Data manipulation and plotting

The visualization of our data was done with R version v3.6.1 (71) and RStudio v1.1.453 (http://www.rstudio.com). All R packages and versions used for general data manipulation and visualization can be found in Table S2.

Supporting Information Figures

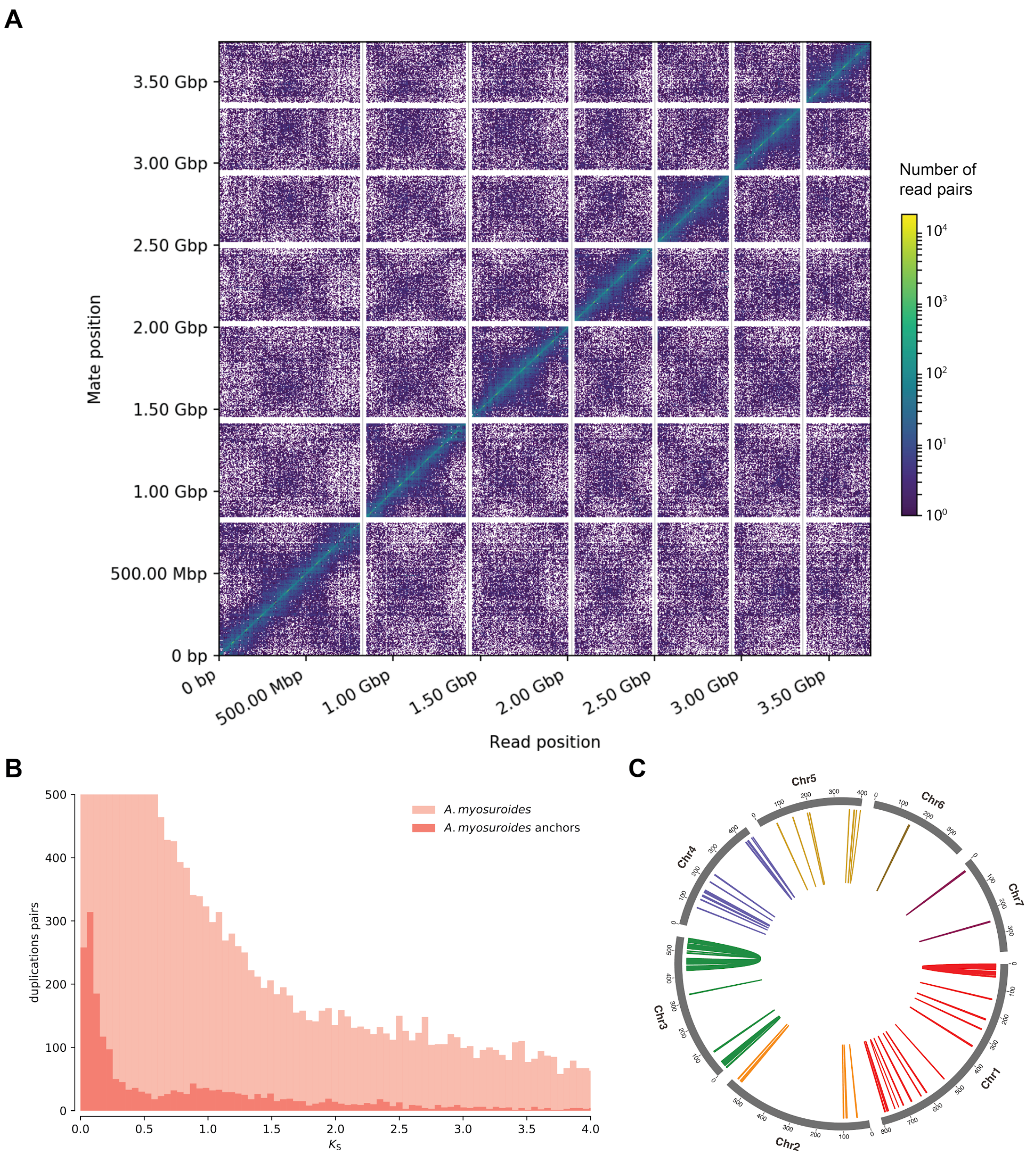

Figure S1. Genome scaffolding and analysis of anchors. **A,** Link density plot of the mapping positions of the first (x-axis) and second read (y-read) in the read pair, grouped into bins. The color of each square indicates the number of read pairs in that bin. Scaffolds < 1 Mb are excluded. **B,** K_S distributions for all paralogs within the *A. myosuroides* (light color) and for the paralogs retained in collinear regions, also known as anchors (dark color). **C,** Circos plot of the *A. myosuroides* genome, with colored lines connecting anchor pairs (genes in the collinear regions) with K_S < 0.5 (Dataset S1).

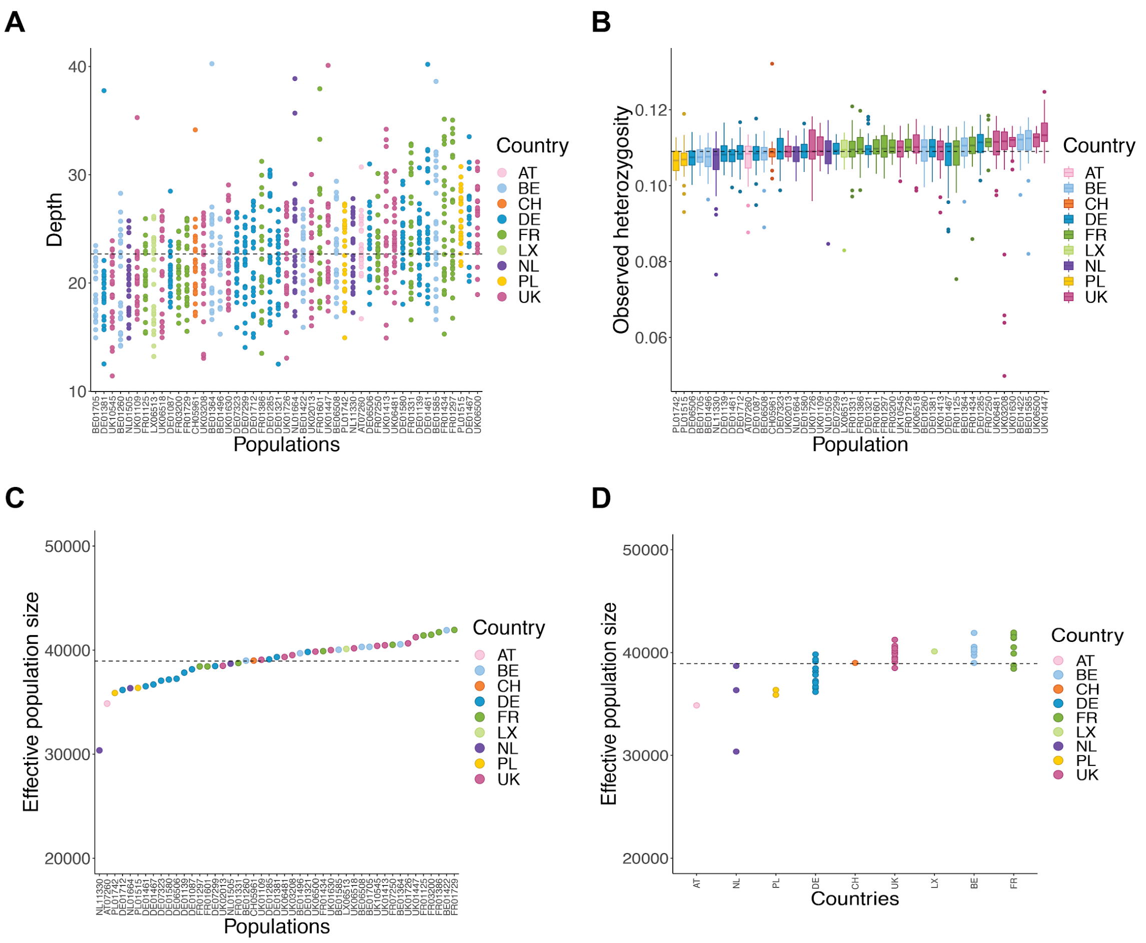

Figure S2. Basic statistics of the ddRAD-Seq dataset and diversity metrics. Colors reflect country-specific origin of the populations. **A,** Sequencing depth. **B,** Observed SNP heterozygosity. **C,** Effective population sizes. Mean= 38,912 individuals (dashed line) **D,** Effective population sizes ordered by countries. Tukey's HSD test showed a significant difference between the mean effective population size of DE and UK ($p<0.03$), DE and BE ($p<0.03$), DE and FR ($p<0.01$). Austria (AT), Belgium (BE), Switzerland (CH), Germany (DE), France (FR), Luxembourg (LX), Netherlands (NL), Poland (PL), United Kingdom (UK).

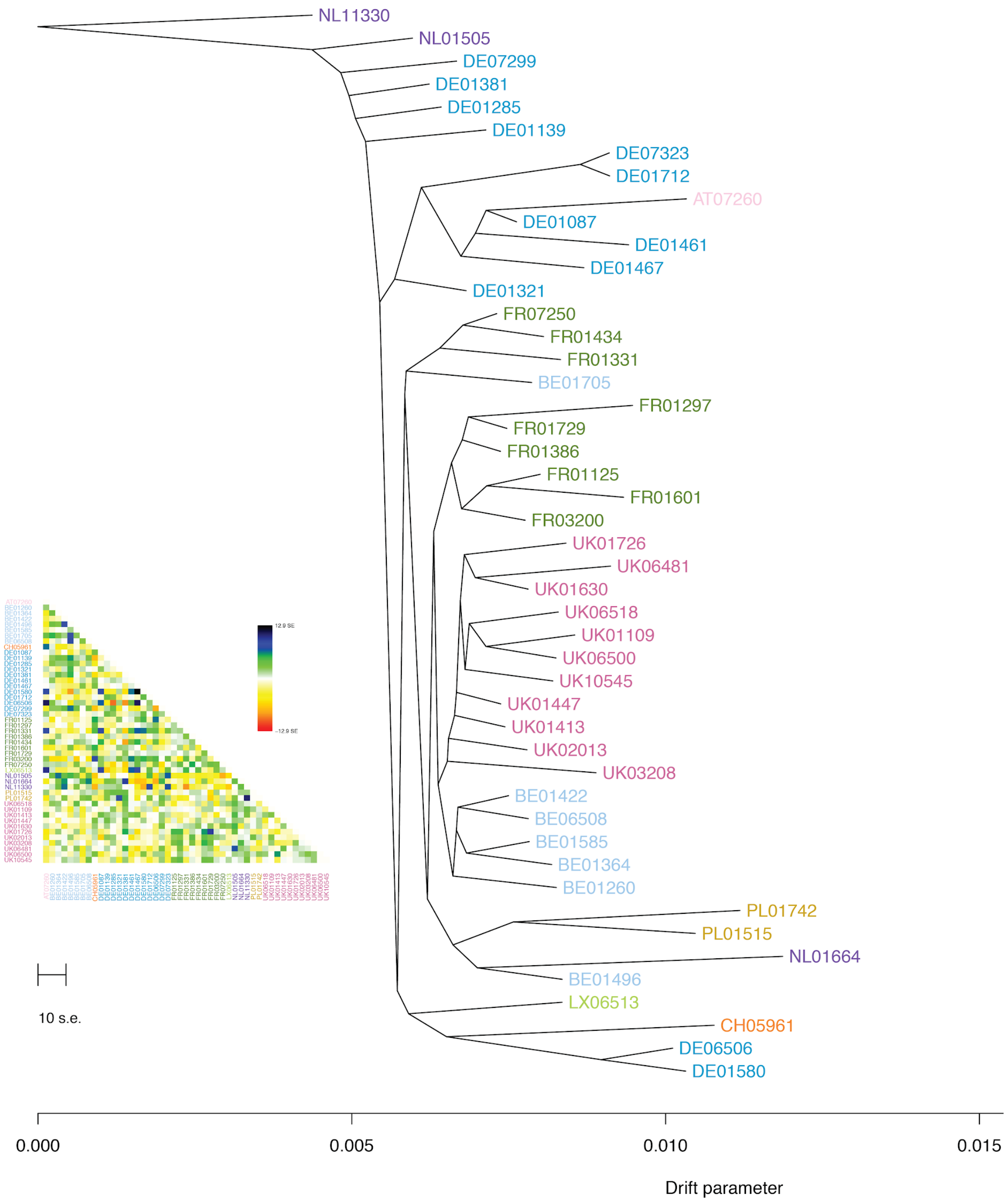

Figure S3. Treemix plot of the relationship of populations with residuals. Colors reflect the country-specific origin of the populations. Austria (AT), Belgium (BE), Switzerland (CH), Germany (DE), France (FR), Luxembourg (LX), Netherlands (NL), Poland (PL), United Kingdom (UK).

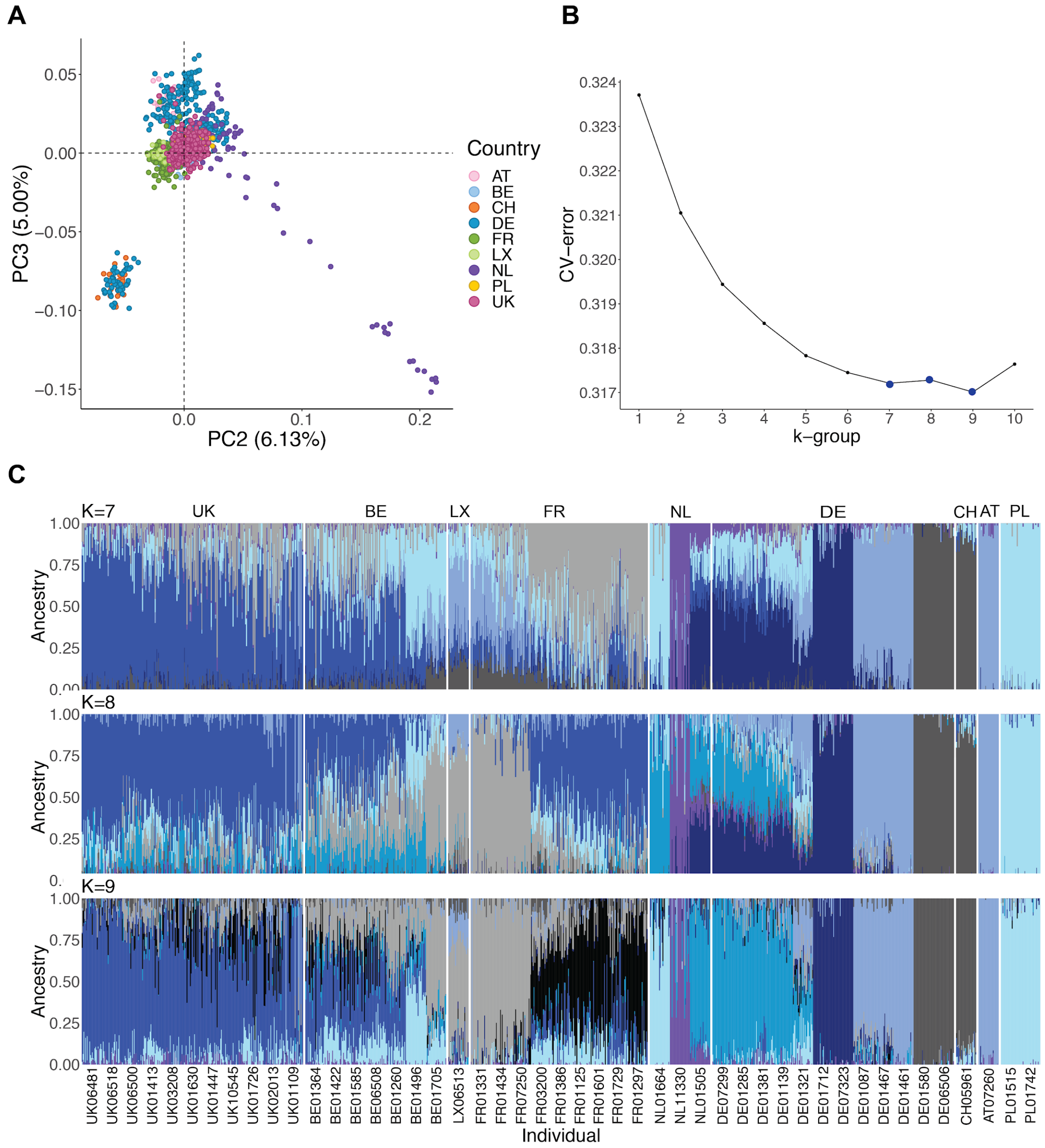

Figure S4. Population structure analysis. **A,** Second and third eigenvectors of the principal component analysis (PCA). The genetic variance of the second and third component is shown in brackets. Colors reflect country-specific origin of the populations. **B,** Cross validation error as a function of K of the admixture analysis. **C**, Admixture proportions with ancestry groups of K=9, K=7 and K=8. Austria (AT), Belgium (BE), Switzerland (CH), Germany (DE), France (FR), Luxembourg (LX), Netherlands (NL), Poland (PL), United Kingdom (UK).

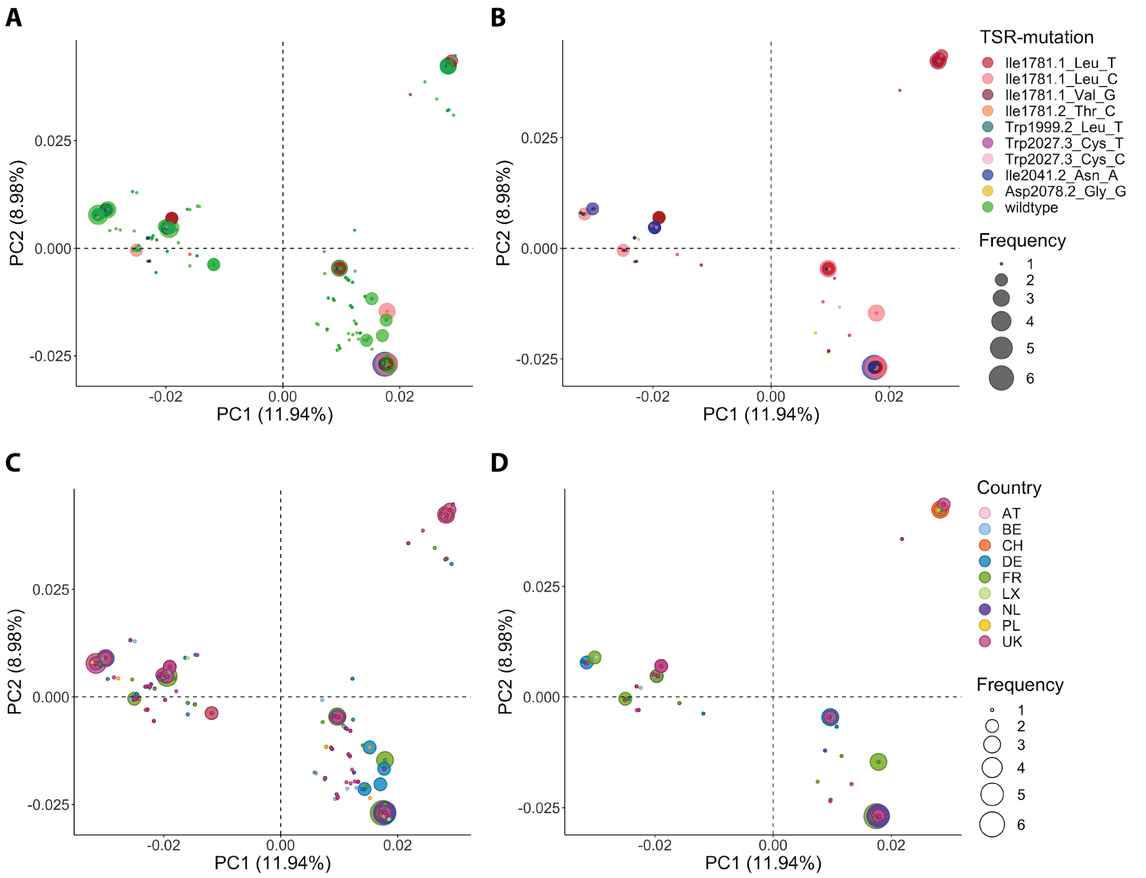

Figure S5. ACCase haplotype principal component analysis (PCA). Eigenvectors of the first two components are shown. **A,** Target-site-resistance (TSR) annotation of all existing haplotypes including wildtype haplotypes. **B,** Only TSR haplotypes. **C,** Country-specific coloring of all existing haplotypes. **D,** Country-specific coloring of exclusively TSR haplotypes. The values in brackets show the explained variance. Austria (AT), Belgium (BE), Switzerland (CH), Germany (DE), France (FR), Luxembourg (LX), Netherlands (NL), Poland (PL), United Kingdom (UK).

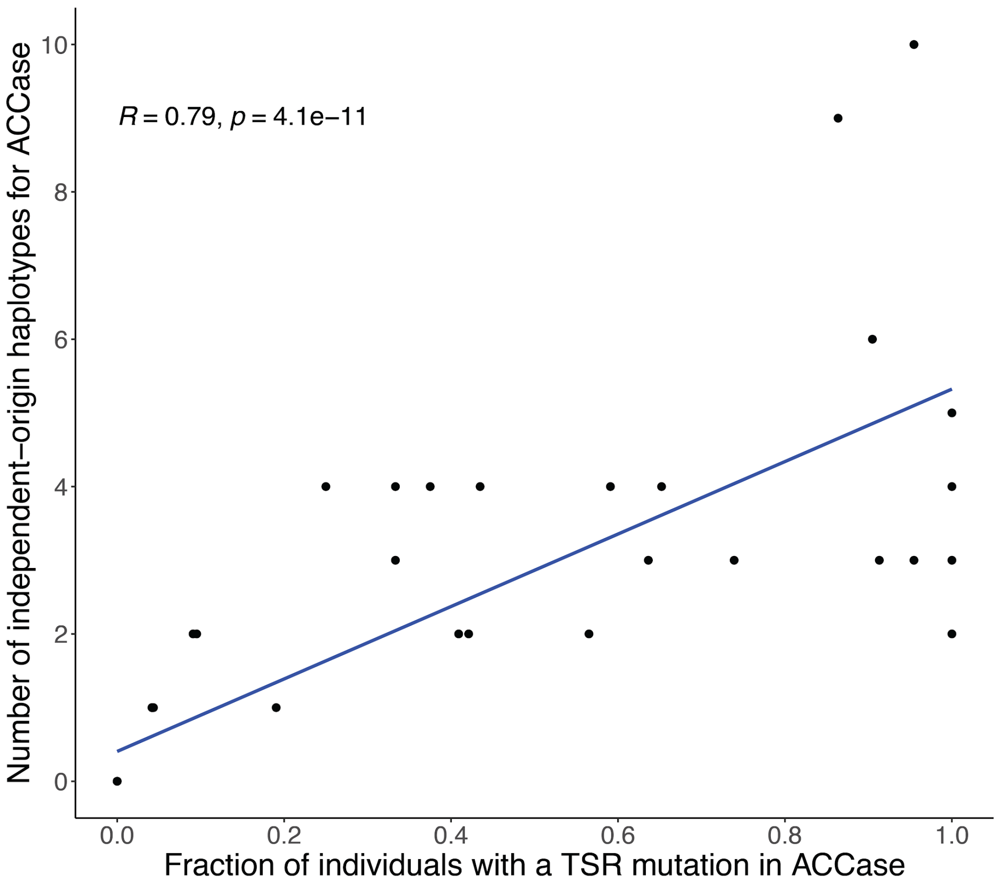

Figure S6. Correlation between the fraction of individuals with TSR mutations and the number of TSR haplotypes for the *ACCase* gene. Every dot represents a population.

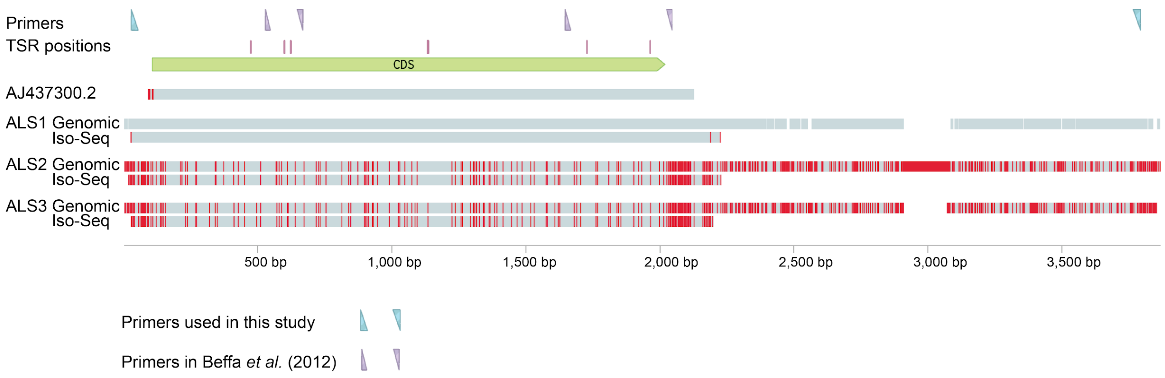

Figure S7. *ALS* copies in *A. myosuroides* genome. Multiple alignment performed with Clustal Omega (72) between the widely studied *ALS* GenBank entry of *A. myosuroides* AJ437300.2 (60), three genomic loci encoding *ALS* genes, and three representative Iso-Seq reads (each with an average read quality of q93) corresponding to each of the three Iso-Seq clusters determined by pbaa (https://github.com/PacificBiosciences/pbAA) with data from all five tissues combined. Indicated are also the positions of the seven known TSR mutations in *ALS*, the primers used in this study to selectively amplify *ALS1*, and the two pairs of primers commonly used to genotype TSRs Pro197 and Ala205 (first pair), and Trp574 and Ser653 (second pair) (73).

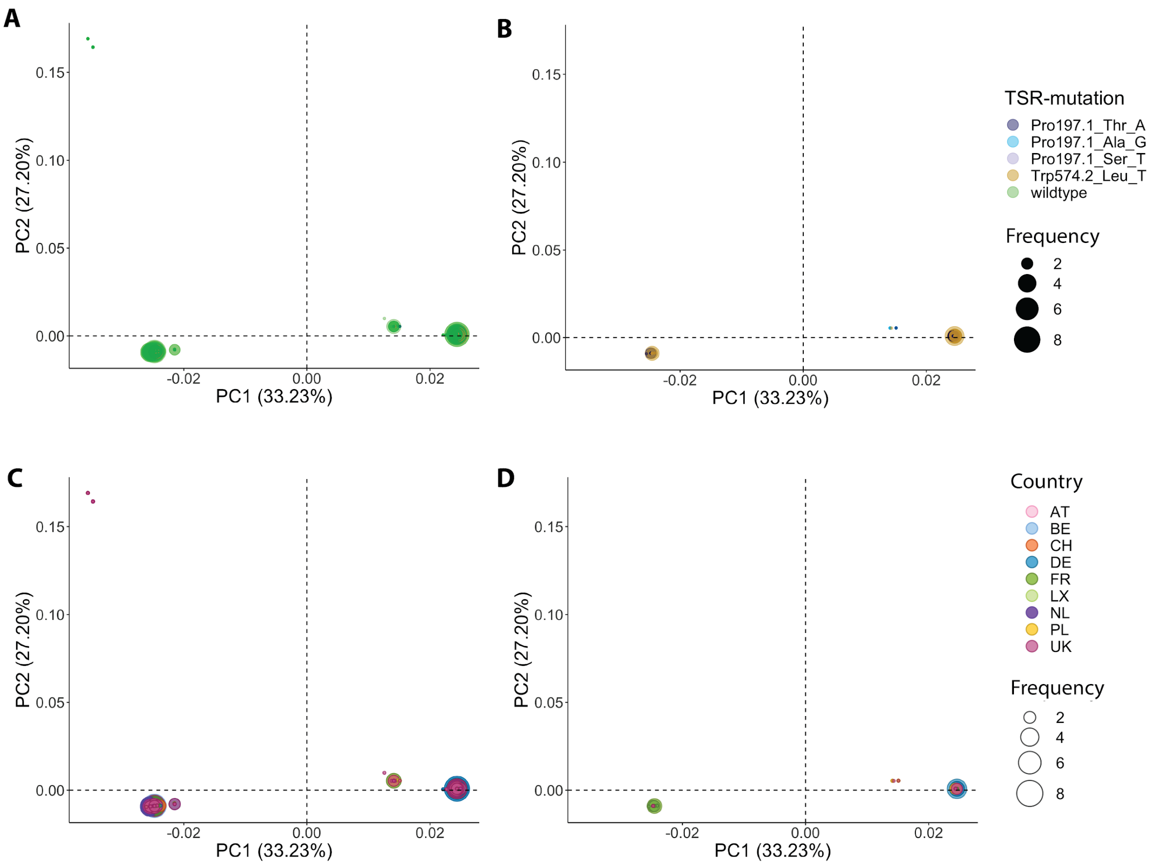

Figure S8. *ALS* haplotype principal component analysis (PCA). Eigenvectors of the first two components are shown. **A,** Target-site-resistance (TSR) annotation of all existing haplotypes including wildtype haplotypes. **B,** Only TSR haplotypes. **C,** Country-specific coloring of all existing haplotypes. **D,** Country-specific coloring of exclusively TSR haplotypes. The values in brackets show the explained variance. Austria (AT), Belgium (BE), Switzerland (CH), Germany (DE), France (FR), Luxembourg (LX), Netherlands (NL), Poland (PL), United Kingdom (UK).

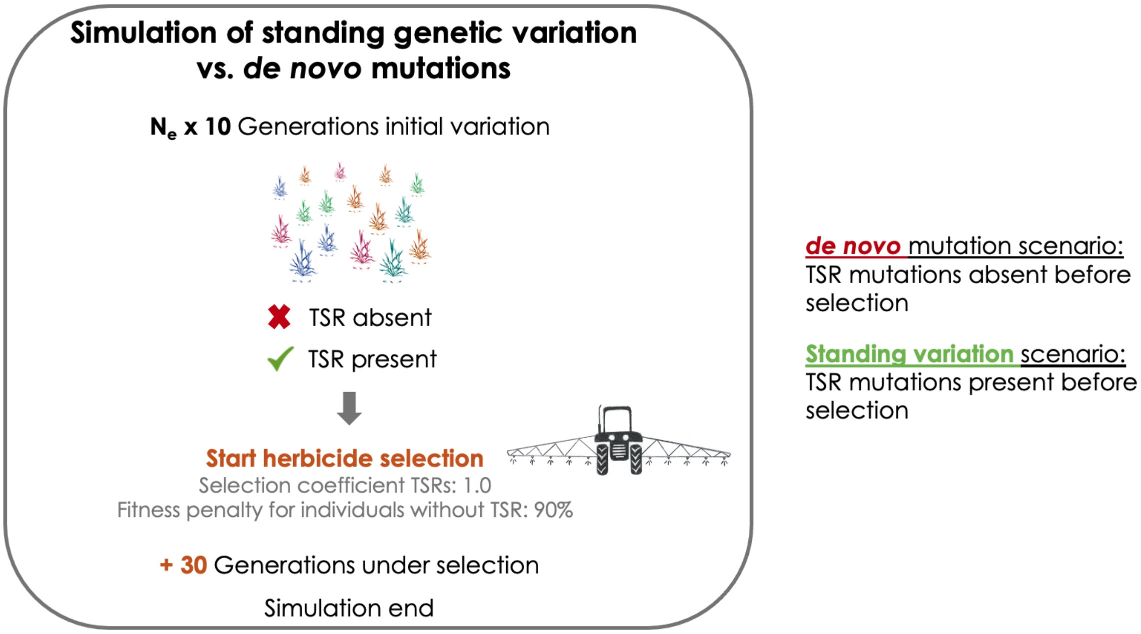

Figure S9. Simulation of herbicide resistance evolution. Visualization of the SLiM simulation model and the two scenarios for the origin of TSR mutation: TSR mutations emerging from standing genetic variation if they were present before the start of herbicide selection, or from *de novo* mutation if they appeared after herbicide selection. The model was run using the intron/exon structure of the *ACCase* locus as a template and without it. For the model with introns/exons, mutations in introns and non-coding regions were considered to be neutral, while exons had a ratio of 0.25/0.75 (neutral/deleterious) mutations according to Messer and Petrov (67), with selection coefficients (s) for deleterious mutations drawn from a gamma distribution with E[s] = -0.000154 and a shape parameter of 0.245 (68). For the latter, all mutations were considered to be neutral. Furthermore, we simulated two N_e values: 42,000 and 84,000 individuals.

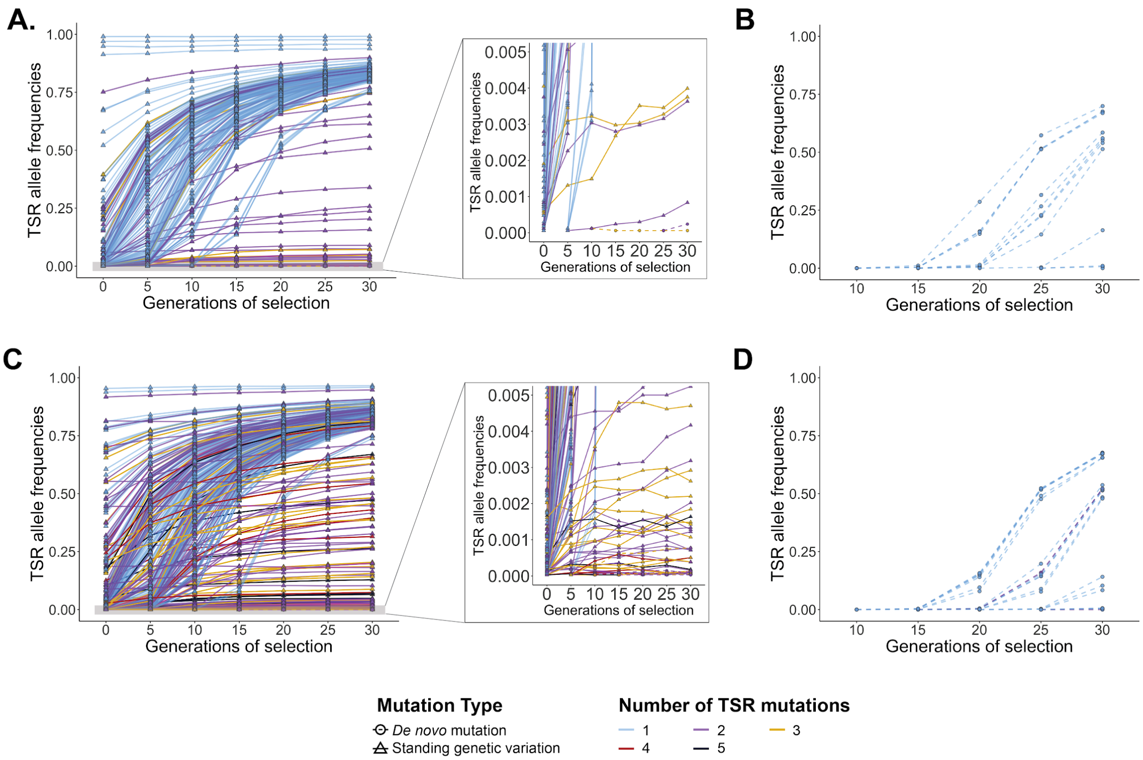

Figure S10. Simulations of expected allele frequencies for TSR alleles arising from standing genetic variation or *de novo* mutation (without intron/exon structure). All mutations were considered to be neutral before the start of selection. Five hundred of one thousand simulation runs are shown for an effective population size (N_e) of (**A, B**) 42,000 individuals and (**C, D**) 84,000 individuals. Continuous lines represent mutations originating from standing genetic variation; *de novo* TSR mutations are shown with dashed lines. Colors indicate the total number of TSR mutations per population. **A, C,** Standing genetic variation scenario, with TSR mutations pre-existing in the populations before herbicide selection. Shown is the increase in TSR allele frequencies under herbicide selection of up to 30 generations, with one herbicide application per generation. The right panel shows a truncated y-axis at 0.005 TSR allele frequencies. **B, D,** *De novo* mutation scenario. Any TSR mutation that might have arisen before the start of selection has been lost again, so that no TSR mutations are present at generation 0 of selection.

A

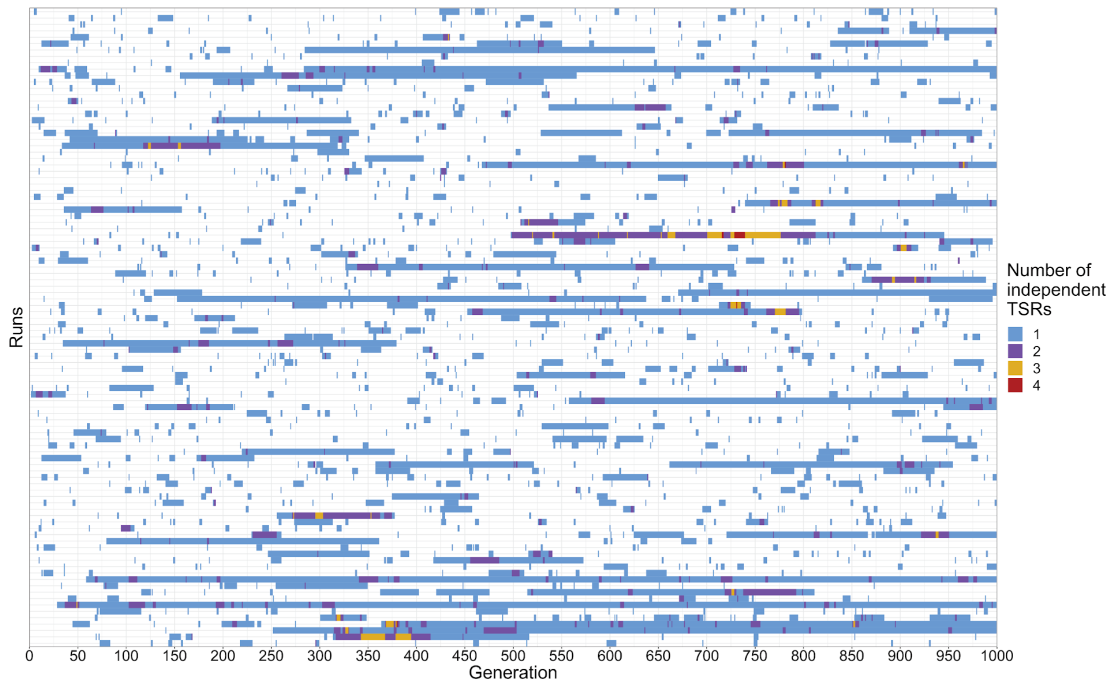

B

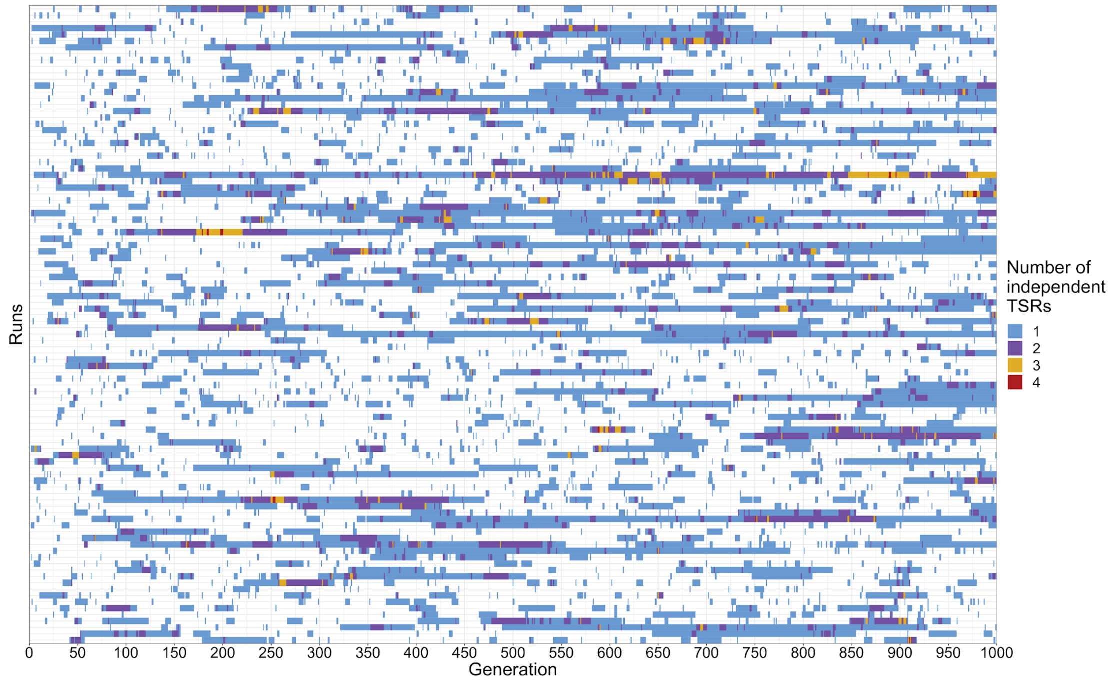

Figure S11. Simulations of TSR abundance under neutrality. SLiM simulations representing 100 independent simulation runs of population evolution for (**A**) 42,000 individuals and (**B**) 84,000 individuals over 1,000 generations under neutrality. The occurrence and loss of TSRs due to genetic drift can be observed. Colors indicate the number of TSRs present in each generation in a given run.

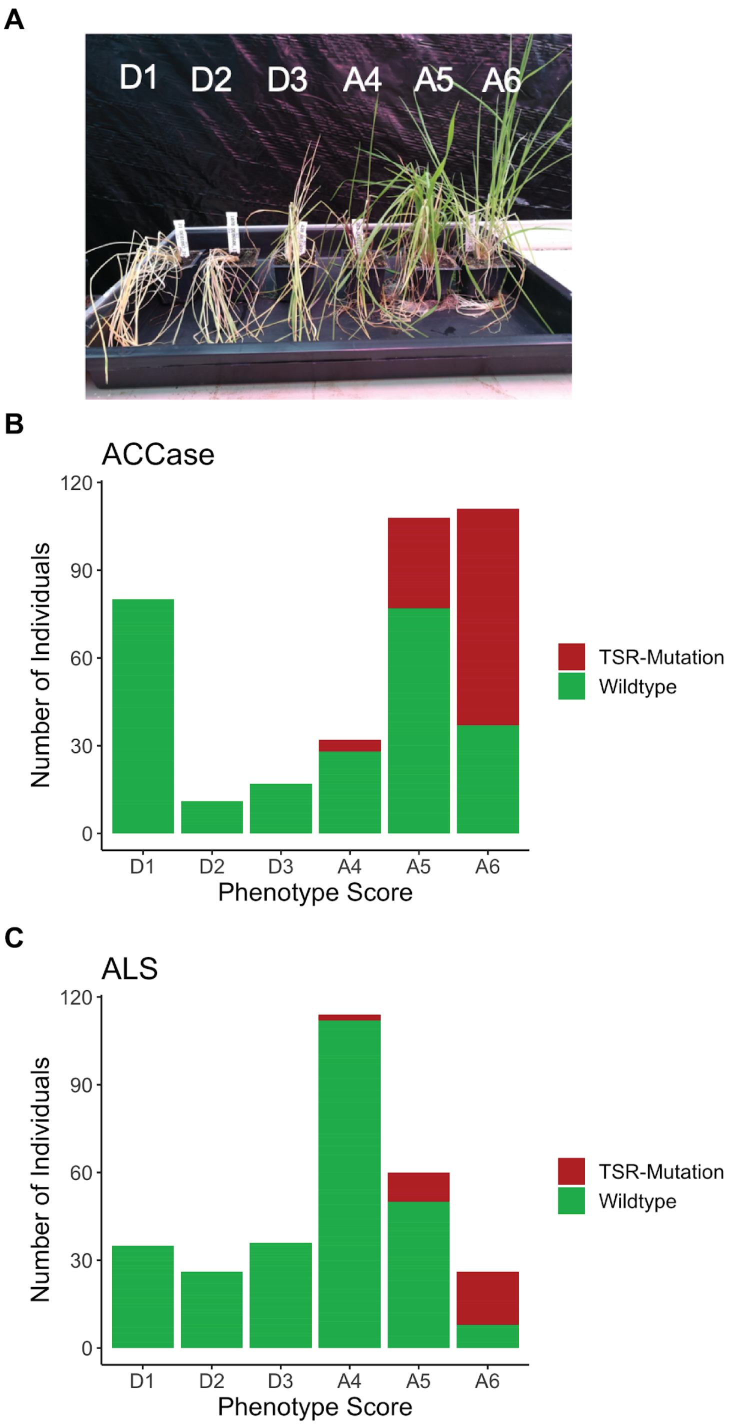

Figure S12. Phenotyping and genotyping of TSR mutations in single individuals. **A,** Phenotype scoring scheme, where the score D1 represents completely dead plants (no green material visible) and the score A6 represents plants without any growth reductions compared to the control plants of the respective population. **B,** Distribution of phenotype scores after treatment with ACCase inhibitor Axial® 50 (pinoxaden + cloquintocet-mexyl). In red, the number of individuals that carry a TSR mutation. In green, wildtype individuals. **C,** Distribution of phenotype scores after treatment with ALS inhibitor Atlantis WG® (mesosulfuron + iodosulfuron).

A

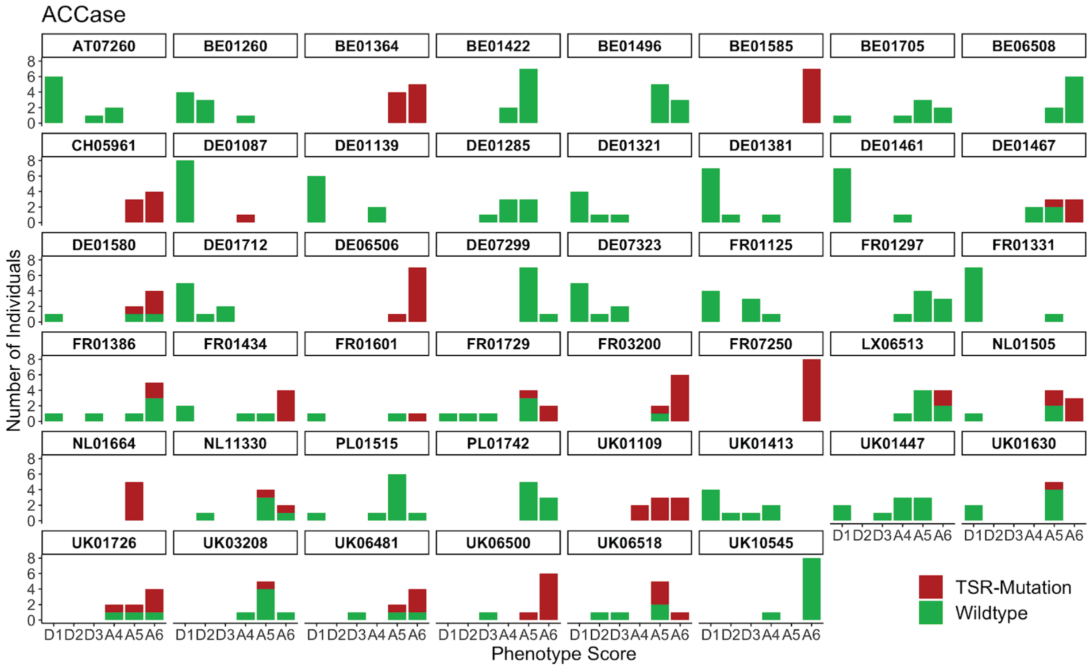

B

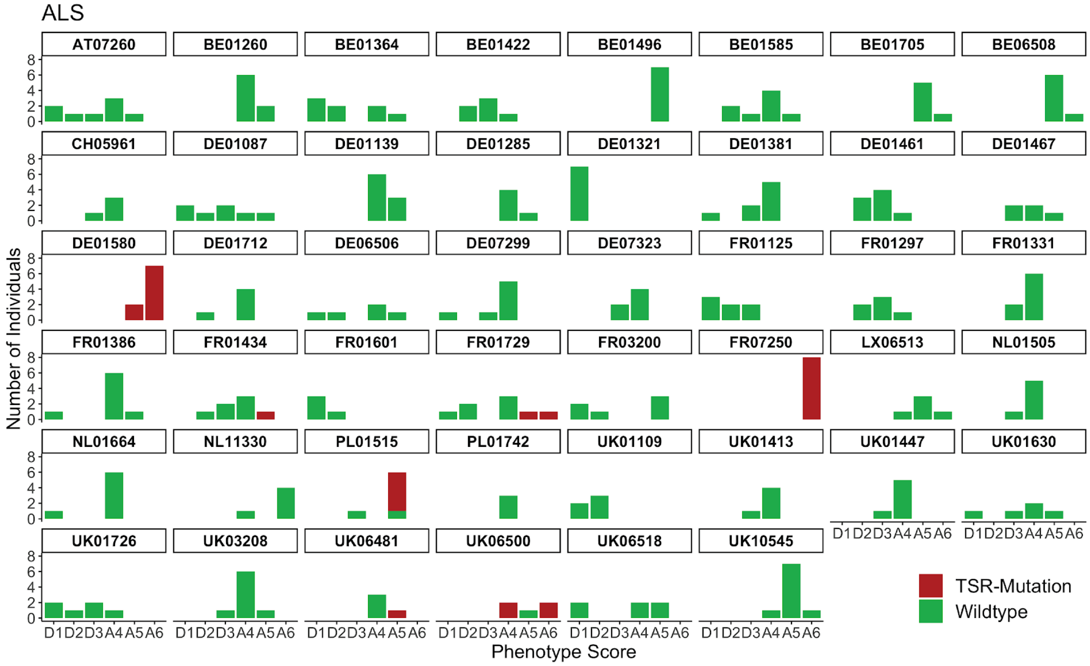

Figure S13. Phenotyping and genotyping of TSR mutations in single individuals per population. Phenotype scoring according to the scheme in Figure S12A, where the score D1 represents completely dead plants (no green material visible) and the score A6 represents plants without any growth reductions compared to the control plants of the respective population. **A,** Distribution of phenotype scores per population after treatment with ACCase inhibitor Axial® 50 (pinoxaden + cloquintocet-mexyl). In red, the number of individuals that carry a TSR mutation. In green, wildtype individuals. **B,** Distribution of phenotype scores after treatment with ALS inhibitor Atlantis WG® (mesosulfuron + iodosulfuron).

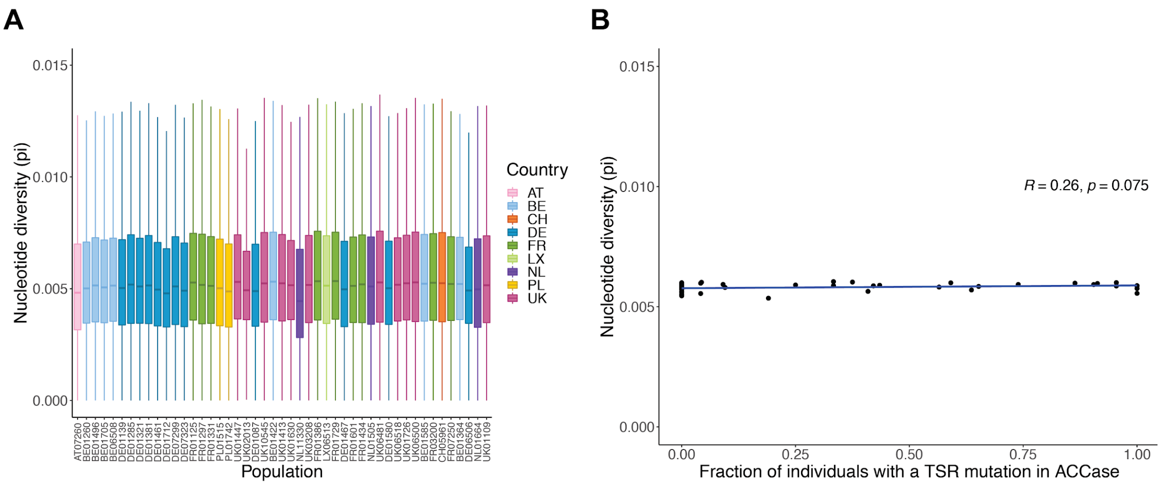

Figure S14. Relationship between nucleotide diversity and fraction of individuals with a TSR mutation in *ACCase* per population. **A,** Nucleotide diversity (pi) per population estimated from ddRAD-Seq data. Populations are sorted, in increasing order, according to the fraction of individuals with a TSR mutation in the *ACCase* gene. Colors reflect the country-specific origin of the populations. Austria (AT), Belgium (BE), Switzerland (CH), Germany (DE), France (FR), Luxembourg (LX), Netherlands (NL), Poland (PL), United Kingdom (UK). **B,** Correlation between the fraction of individuals with TSR mutations in the *ACCase* gene and nucleotide diversity (pi) per population. Every dot represents a population.

Supporting Information Tables

Table S1. TSR and TSR haplotype number per population.

Population	*ACCase*			*ALS*		
	TSR number	TSR haplotype number	Min. indep. haplotype origins	TSR number	TSR haplotypes	Min. indep. haplotype origins
AT07260	0	0	0	0	0	0
BE01260	0	0	0	0	0	0
BE01364	4	5	5	0	0	0
BE01422	1	1	1	0	0	0
BE01496	0	0	0	0	0	0
BE01585	5	7	6	0	0	0
BE01705	0	0	0	0	0	0
BE06508	0	0	0	0	0	0
CH05961	3	4	3	0	0	0
DE01087	1	1	1	0	0	0
DE01139	0	0	0	0	0	0
DE01285	0	0	0	0	0	0
DE01321	0	0	0	0	0	0
DE01381	0	0	0	0	0	0
DE01461	0	0	0	0	0	0
DE01467	2	2	2	0	0	0
DE01580	3	3	3	2	2	2
DE01712	0	0	0	0	0	0
DE06506	3	4	4	0	0	0
DE07299	0	0	0	0	0	0
DE07323	0	0	0	0	0	0
FR01125	0	0	0	0	0	0

FR01297	0	0	0	0	0	0
FR01331	0	0	0	0	0	0
FR01386	3	4	4	1	1	1
FR01434	3	4	4	2	2	2
FR01601	2	2	2	0	0	0
FR01729	3	4	4	1	2	2
FR03200	2	3	3	0	0	0
FR07250	6	10	10	2	3	3
LX06513	2	3	3	0	0	0
NL01505	2	2	2	0	0	0
NL01664	2	2	2	0	0	0
NL11330	1	1	1	0	0	0
PL01515	0	0	0	2	2	2
PL01742	0	0	0	0	0	0
UK01109	2	4	3	0	0	0
UK01413	2	2	2	0	0	0
UK01447	0	0	0	0	0	0
UK01630	2	2	2	0	0	0
UK01726	3	3	3	0	0	0
UK02013	0	0	0	0	0	0
UK03208	2	4	4	0	0	0
UK06481	3	5	4	1	1	1
UK06500	4	9	9	2	3	3
UK06518	3	5	4	0	0	0
UK10545	1	1	1	1	1	1

Table S2. R-packages used for data manipulation and visualization.

Package name and version	Reference
ComplexHeatmap 2.0.0	Gu, 2016 (50)
dplyr 1.0.2	Wickham, 2020 (74)
gdsfmt 1.20.0	Zheng, 2012 (45)
GetoptLong 1.0.4	Gu, 2020 (https://github.com/jokergoo/GetoptLong)
ggplot 3.3.2	Wickham, 2016 (59)
ggpubr 0.4.0	Kassambara, 2020 (https://github.com/kassambara/ggpubr/)
ggtree 1.16.6	Yu, 2017 (57)
ggthemes 4.2.0	Arnold, 2021 (https://github.com/jrnold/ggthemes/)
gtable 0.3.0	Wickham, 2019 (https://github.com/r-lib/gtable)
haplotypes 1.1.2	Aktas, 2020 (https://cran.r-project.org/web/packages/haplotypes/haplotypes.pdf)
patchwork 1.1.0	Pedersen, 2020 (https://github.com/thomasp85/patchwork)
plotly 4.9.2.1	Sievert, 2019 (75)
plyr 1.8.6	Wickham, 2011 (76)
qqman 0.1.4	Turner, 2021 (https://github.com/stephenturner/qqman)
SNPRelate 1.18.1	Zheng, 2012 (45)
stats 3.6.1	The R core team (71)
tibble 3.1.6	Müller,2021 (https://github.com/tidyverse/tibble/)
tidyr 1.1.2	Wickham, 2020 (https://github.com/tidyverse/tidyr/)
tidyverse 1.3.0	Wickham, 2019 (77)
treeio 1.18.1	Wang, 2020 (58)
vcfR 1.12.0	Knaus, 2017 (78)

Legends for Datasets

Dataset S1. Sheet1, List of paralogs retained in collinear regions (anchors), their K_S values, and whether they are part of Figure S1C. **Sheet2,** List of 250 non-redundant *ACCase* haplotypes. **Sheet3,** List of primers used in this study.

Dataset S2. *ACCase* networks and trees for 47 European populations. Haplotype network and maximum likelihood (ML)-tree per population. The color code in all networks and trees shows target-site resistances (TSRs) and wildtype haplotypes in green.

Dataset S3. *ALS* networks and trees for 47 European populations. Haplotype network and maximum likelihood (ML)-tree per population. The color code in all networks and trees shows target-site resistances (TSRs) and wildtype haplotypes in green.

Supporting Information References

1. S. Picelli, *et al.*, Tn5 transposase and tagmentation procedures for massively scaled sequencing projects. *Genome Res.* **24**, 2033–2040 (2014).

2. C. Délye, X.-Q. Zhang, S. Michel, A. Matéjicek, S. B. Powles, Molecular bases for sensitivity to acetyl-coenzyme A carboxylase inhibitors in black-grass. *Plant Physiol.* **137**, 794–806 (2005).

3. H. Xu, *et al.*, Mutations at codon position 1999 of acetyl-CoA carboxylase confer resistance to ACCase-inhibiting herbicides in Japanese foxtail (Alopecurus japonicus). *Pest Manag. Sci.* **70**, 1894–1901 (2014).

4. J. Doležel, J. Greilhuber, S. Lucretti, A. Meister, Plant genome size estimation by flow cytometry: inter-laboratory comparison. *Annals of Botany* **82**, 17–26 (1998).

5. J. Dolezel, J. Bartos, Plant DNA flow cytometry and estimation of nuclear genome size. *Ann. Bot.* **95**, 99–110 (2005).

6. R. Workman, *et al.*, High Molecular Weight DNA Extraction from Recalcitrant Plant Species for Third Generation Sequencing v1 (protocols.io.4vbgw2n) https:/doi.org/10.17504/protocols.io.4vbgw2n.

7. H. Yaffe, *et al.*, LogSpin: a simple, economical and fast method for RNA isolation from infected or healthy plants and other eukaryotic tissues. *BMC Res. Notes* **5**, 45 (2012).

8. A. Acosta-Maspons, I. González-Lemes, A. A. Covarrubias, Improved protocol for isolation of high-quality total RNA from different organs of Phaseolus vulgaris L. *Biotechniques* **66**, 96–98 (2019).

9. E. Lieberman-Aiden, *et al.*, Comprehensive mapping of long-range interactions reveals folding principles of the human genome. *Science* **326**, 289–293 (2009).

10. C.-S. Chin, *et al.*, Phased diploid genome assembly with single-molecule real-time sequencing. *Nat. Methods* **13**, 1050–1054 (2016).

11. D. Guan, *et al.*, Identifying and removing haplotypic duplication in primary genome assemblies. *Bioinformatics* **36**, 2896–2898 (2020).

12. N. H. Putnam, *et al.*, Chromosome-scale shotgun assembly using an in vitro method for long-range linkage. *Genome Res.* **26**, 342–350 (2016).

13. H. Li, Aligning sequence reads, clone sequences and assembly contigs with BWA-MEM. *arXiv [q-bio.GN]* (2013).

14. S. Ou, *et al.*, Benchmarking transposable element annotation methods for creation of a streamlined, comprehensive pipeline. *Genome Biol.* **20**, 275 (2019).

15. T. D. Wu, C. K. Watanabe, GMAP: a genomic mapping and alignment program for mRNA and EST sequences. *Bioinformatics* **21**, 1859–1875 (2005).

16. D. Kim, J. M. Paggi, C. Park, C. Bennett, S. L. Salzberg, Graph-based genome alignment and genotyping with HISAT2 and HISAT-genotype. *Nat. Biotechnol.* **37**, 907–915 (2019).

17. S. Kovaka, *et al.*, Transcriptome assembly from long-read RNA-seq alignments with StringTie2. *Genome Biol.* **20**, 278 (2019).

18. C. Trapnell, *et al.*, Transcript assembly and quantification by RNA-Seq reveals unannotated transcripts and isoform switching during cell differentiation. *Nat. Biotechnol.* **28**, 511–515 (2010).

19. S. F. Altschul, W. Gish, W. Miller, E. W. Myers, D. J. Lipman, Basic local alignment search tool. *J. Mol. Biol.* **215**, 403–410 (1990).

20. UniProt Consortium, UniProt: the universal protein knowledgebase in 2021. *Nucleic Acids Res.* **49**, D480–D489 (2021).

21. T. Brůna, K. J. Hoff, A. Lomsadze, M. Stanke, M. Borodovsky, BRAKER2: automatic eukaryotic genome annotation with GeneMark-EP+ and AUGUSTUS supported by a protein database. *NAR Genom Bioinform* **3**, lqaa108 (2021).

22. M. Van Bel, *et al.*, PLAZA 4.0: an integrative resource for functional, evolutionary and comparative plant genomics. *Nucleic Acids Res.* **46**, D1190–D1196 (2018).

23. G. S. C. Slater, E. Birney, Automated generation of heuristics for biological sequence comparison. *BMC Bioinformatics* **6**, 31 (2005).

24. B. J. Haas, *et al.*, Automated eukaryotic gene structure annotation using EVidenceModeler and the Program to Assemble Spliced Alignments. *Genome Biol.* **9**, R7 (2008).

25. F. A. Simão, R. M. Waterhouse, P. Ioannidis, E. V. Kriventseva, E. M. Zdobnov, BUSCO: assessing genome assembly and annotation completeness with single-copy orthologs. *Bioinformatics* **31**, 3210–3212 (2015).

26. P. Jones, *et al.*, InterProScan 5: genome-scale protein function classification. *Bioinformatics* **30**, 1236–1240 (2014).

27. A. Zwaenepoel, Y. Van de Peer, wgd-simple command line tools for the analysis of ancient whole-genome duplications. *Bioinformatics* **35**, 2153–2155 (2019).

28. K. Katoh, D. M. Standley, MAFFT multiple sequence alignment software version 7: improvements in performance and usability. *Mol. Biol. Evol.* **30**, 772–780 (2013).

29. Z. Yang, PAML 4: phylogenetic analysis by maximum likelihood. *Mol. Biol. Evol.* **24**, 1586–1591 (2007).

30. S. Proost, *et al.*, i-ADHoRe 3.0--fast and sensitive detection of genomic homology in extremely large data sets. *Nucleic Acids Res.* **40**, e11 (2012).

31. A. H. Paterson, J. E. Bowers, B. A. Chapman, Ancient polyploidization predating divergence of the cereals, and its consequences for comparative genomics. *Proc. Natl. Acad. Sci. U. S. A.* **101**, 9903–9908 (2004).

32. International Brachypodium Initiative, Genome sequencing and analysis of the model grass Brachypodium distachyon. *Nature* **463**, 763–768 (2010).

33. H. Tang, *et al.*, Synteny and collinearity in plant genomes. *Science* **320**, 486–488 (2008).

34. C. Chen, *et al.*, TBtools: An Integrative Toolkit Developed for Interactive Analyses of Big Biological Data. *Mol. Plant* **13**, 1194–1202 (2020).

35. P. L. M. Lang, *et al.*, Hybridization ddRAD-sequencing for population genomics of nonmodel plants using highly degraded historical specimen DNA. *Mol. Ecol. Resour.* **20**, 1228–1247 (2020).

36. N. Rohland, D. Reich, Cost-effective, high-throughput DNA sequencing libraries for multiplexed target capture. *Genome Res.* **22**, 939–946 (2012).

37. M. Martin, Cutadapt removes adapter sequences from high-throughput sequencing reads. *EMBnet.journal* **17**, 10–12 (2011).

38. T. Magoč, S. L. Salzberg, FLASH: fast length adjustment of short reads to improve genome assemblies. *Bioinformatics* **27**, 2957–2963 (2011).

39. H. Li, *et al.*, The Sequence Alignment/Map format and SAMtools. *Bioinformatics* **25**, 2078–2079 (2009).

40. G. A. Van der Auwera, *et al.*, From FastQ data to high confidence variant calls: the Genome Analysis Toolkit best practices pipeline. *Curr. Protoc. Bioinformatics* **43**, 11.10.1–11.10.33 (2013).

41. P. Danecek, *et al.*, The variant call format and VCFtools. *Bioinformatics* **27**, 2156–2158 (2011).

42. J. B. Puritz, C. M. Hollenbeck, J. R. Gold, dDocent: a RADseq, variant-calling pipeline designed for population genomics of non-model organisms. *PeerJ* **2**, e431 (2014).

43. A. M. Kozlov, D. Darriba, T. Flouri, B. Morel, A. Stamatakis, RAxML-NG: a fast, scalable and user-friendly tool for maximum likelihood phylogenetic inference. *Bioinformatics* **35**, 4453–4455 (2019).

44. I. Letunic, P. Bork, Interactive Tree Of Life v2: online annotation and display of phylogenetic trees made easy. *Nucleic Acids Res.* **39**, W475–8 (2011).

45. X. Zheng, *et al.*, A high-performance computing toolset for relatedness and principal component analysis of SNP data. *Bioinformatics* **28**, 3326–3328 (2012).

46. C. C. Chang, *et al.*, Second-generation PLINK: rising to the challenge of larger and richer datasets. *Gigascience* **4**, 7 (2015).

47. D. H. Alexander, K. Lange, Enhancements to the ADMIXTURE algorithm for individual ancestry estimation. *BMC Bioinformatics* **12**, 246 (2011).

48. J. K. Pickrell, J. K. Pritchard, Inference of population splits and mixtures from genome-wide allele frequency data. *PLoS Genet.* **8**, e1002967 (2012).

49. N. C. Rochette, A. G. Rivera-Colón, J. M. Catchen, Stacks 2: Analytical methods for paired-end sequencing improve RADseq-based population genomics. *Mol. Ecol.* **28**, 4737–4754 (2019).

50. Z. Gu, R. Eils, M. Schlesner, Complex heatmaps reveal patterns and correlations in multidimensional genomic data. *Bioinformatics* **32**, 2847–2849 (2016).

51. T. S. Korneliussen, A. Albrechtsen, R. Nielsen, ANGSD: Analysis of Next Generation Sequencing Data. *BMC Bioinformatics* **15**, 356 (2014).

52. N. Yang, *et al.*, Contributions of Zea mays subspecies mexicana haplotypes to modern maize. *Nat. Commun.* **8**, 1874 (2017).

53. H. Li, Minimap2: pairwise alignment for nucleotide sequences. *Bioinformatics* **34**, 3094–3100 (2018).

54. P. Cingolani, *et al.*, A program for annotating and predicting the effects of single nucleotide

polymorphisms, SnpEff: SNPs in the genome of Drosophila melanogaster strain w1118; iso-2; iso-3. *Fly* **6**, 80–92 (2012).

55. H. E. L. Lischer, L. Excoffier, PGDSpider: an automated data conversion tool for connecting population genetics and genomics programs. *Bioinformatics* **28**, 298–299 (2012).

56. J. W. Leigh, D. Bryant, popart : full-feature software for haplotype network construction. *Methods Ecol. Evol.* **6**, 1110–1116 (2015).

57. G. Yu, D. K. Smith, H. Zhu, Y. Guan, T. T. Lam, Ggtree : An r package for visualization and annotation of phylogenetic trees with their covariates and other associated data. *Methods Ecol. Evol.* **8**, 28–36 (2017).

58. L.-G. Wang, *et al.*, Treeio: An R Package for Phylogenetic Tree Input and Output with Richly Annotated and Associated Data. *Mol. Biol. Evol.* **37**, 599–603 (2020).

59. H. Wickham, *ggplot2: Elegant Graphics for Data Analysis* (Springer, Cham, 2016).

60. C. Délye, K. Boucansaud, A molecular assay for the proactive detection of target site-based resistance to herbicides inhibiting acetolactate synthase in Alopecurus myosuroides. *Weed Res.* **48**, 97–101 (2008).

61. Z. Zhang, S. Schwartz, L. Wagner, W. Miller, A greedy algorithm for aligning DNA sequences. *J. Comput. Biol.* **7**, 203–214 (2000).

62. J. Hermisson, P. S. Pennings, Soft sweeps and beyond: understanding the patterns and probabilities of selection footprints under rapid adaptation. *Methods Ecol. Evol.* **8**, 700–716 (2017).

63. P. W. Messer, D. A. Petrov, Population genomics of rapid adaptation by soft selective sweeps. *Trends Ecol. Evol.* **28**, 659–669 (2013).

64. P. J. Tranel, T. R. Wright, Resistance of weeds to ALS-inhibiting herbicides: what have we learned? *Weed Sci.* **50**, 700–712 (2002).

65. C. Délye, K. K Boucansaud, A molecular assay for the proactive detection of target site-based resistance to herbicides inhibiting acetolactate synthase in Alopecurus myosuroides. *European Weed Research Society Weed Research* **48**, 97–101 (2007).

66. B. C. Haller, P. W. Messer, SLiM 3: Forward Genetic Simulations Beyond the Wright-Fisher Model. *Mol. Biol. Evol.* **36**, 632–637 (2019).

67. P. W. Messer, D. A. Petrov, Frequent adaptation and the McDonald-Kreitman test. *Proc. Natl. Acad. Sci. U. S. A.* **110**, 8615–8620 (2013).

68. C. D. Huber, A. Durvasula, A. M. Hancock, K. E. Lohmueller, Gene expression drives the evolution of dominance. *Nat. Commun.* **9**, 2750 (2018).

69. E. Bauer, *et al.*, Intraspecific variation of recombination rate in maize. *Genome Biol.* **14**, R103 (2013).

70. D. K. Foster, P. Ward, R. T. Hewson, Selective grass-weed control in wheat and barley based on the safener fenchlorazole-ethyl in *BRIGHTON CROP PROTECTION CONFERENCE WEEDS*, (BRIT CROP PROTECTION COUNCIL, 1993), pp. 1267–1267.

71. R. C. Team, R: a language and environment for statistical computing computer program, version 3.5. 0 (2018).

72. F. Sievers, *et al.*, Fast, scalable generation of high-quality protein multiple sequence alignments using Clustal Omega. *Mol. Syst. Biol.* **7**, 539 (2011).

73. R. Beffa, *et al.*, Weed resistance diagnostic technologies to detect herbicide resistance in cereal-growing areas. A review. *Julius-Kühn-Archiv* **434**, 75–80 (2012).

74. H. Wickham, R. François, L. Henry, K. Müller, A Grammar of Data Manipulation [R package dplyr version 1.0.2] (2020) (November 17, 2021).

75. C. Sievert, Interactive web-based data visualization with R, plotly, and shiny (2020).

76. H. Wickham, The split-apply-combine strategy for data analysis. *J. Stat. Softw.* **40** (2011).

77. H. Wickham, *et al.*, Welcome to the tidyverse. *J. Open Source Softw.* **4**, 1686 (2019).

78. B. J. Knaus, N. J. Grünwald, vcfr: a package to manipulate and visualize variant call format data in R. *Mol. Ecol. Resour.* **17**, 44–53 (2017).
